# Adjuvant Oral Uracil-Tegafur with Leucovorin for Colorectal Cancer Liver Metastases: A Randomized Controlled Trial

**DOI:** 10.1371/journal.pone.0162400

**Published:** 2016-09-02

**Authors:** Kiyoshi Hasegawa, Akio Saiura, Tadatoshi Takayama, Shinichi Miyagawa, Junji Yamamoto, Masayoshi Ijichi, Masanori Teruya, Fuyo Yoshimi, Seiji Kawasaki, Hiroto Koyama, Masaru Oba, Michiro Takahashi, Nobuyuki Mizunuma, Yutaka Matsuyama, Toshiaki Watanabe, Masatoshi Makuuchi, Norihiro Kokudo

**Affiliations:** 1 Hepato-Biliary-Pancreatic Surgery Division, Department of Surgery, Graduate School of Medicine, University of Tokyo, Tokyo, Japan; 2 Department of Gastrointestinal Surgery, Cancer Institute Hospital, Japanese Foundation for Cancer Research, Tokyo, Japan; 3 Department of Digestive Surgery, Nihon University School of Medicine, Tokyo, Japan; 4 First Department of Surgery, Shinshu University School of Medicine, Matsumoto, Japan; 5 Department of Surgery, National Defense Medical College, Tokorozawa, Japan; 6 Department of Surgery, JCHO Tokyo Yamate Medical Center, Tokyo, Japan; 7 Department of Surgery, Showa General Hospital, Tokyo, Japan; 8 Department of Surgery, Ibaraki Prefectural Central Hospital and Cancer Center, Ibaraki, Japan; 9 Department of Hepatobiliary-Pancreatic Surgery, Juntendo University School of Medicine, Tokyo, Japan; 10 Department of Surgery, JCHO Tokyo Takanawa Hospital, Tokyo, Japan; 11 Department of Gastroenterology, Cancer Institute Hospital, Japanese Foundation for Cancer Research, Tokyo, Japan; 12 Department of Biostatistics, School of Public Health University of Tokyo, Tokyo, Japan; 13 Department of Surgical Oncology, Graduate School of Medicine, University of Tokyo, Tokyo, Japan; 14 Department of Hepato-Biliary-Pancreatic Surgery, Japanese Red Cross Medical Center, Tokyo, Japan; Taipei Veterans General Hospital, TAIWAN

## Abstract

**Background:**

The high recurrence rate after surgery for colorectal cancer liver metastasis (CLM) remains a crucial problem. The aim of this trial was to evaluate the efficacy of adjuvant therapy with uracil-tegafur and leucovorin (UFT/LV).

**Methods:**

In the multicenter, open-label, phase III trial, patients undergoing curative resection of CLM were randomly assigned in a 1:1 ratio to either the UFT/LV group or surgery alone group. The UFT/LV group orally received 5 cycles of adjuvant UFT/LV (UFT 300mg/m^2^ and LV 75mg/day for 28 days followed by a 7-day rest per cycle). The primary endpoint was recurrence-free survival (RFS). Secondary endpoints included overall survival (OS).

**Results:**

Between February 2004 and December 2010, 180 patients (90 in each group) were enrolled into the study. Of these, 3 patients (2 in the UFT/LV group and 1 in the surgery alone group) were excluded from the efficacy analysis. Median follow-up was 4.76 (range, 0.15–9.84) years. The RFS rate at 3 years was higher in the UFT/LV group (38.6%, n = 88) than in the surgery alone group (32.3%, n = 89). The median RFS in the UFT/LV and surgery alone groups were 1.45 years and 0.70 years, respectively. UFT/LV significantly prolonged the RFS compared with surgery alone with the hazard ratio of 0.56 (95% confidence interval, 0.38–0.83; P = 0.003). The hazard ratio for death of the UFT/LV group against the surgery alone group was not significant (0.80; 95% confidence interval, 0.48–1.35; P = 0.409).

**Conclusion:**

Adjuvant therapy with UFT/LV effectively prolongs RFS after hepatic resection for CLM and can be recommended as an alternative choice.

**Trial Registration:**

UMIN Clinical Trials Registry C000000013

## Introduction

Although hepatic resection is the standard treatment for resectable colorectal cancer liver metastases (CLM), relapse is still common, which occurs in 70% to 80% of patients at 5 years even after curative hepatic resection [1, 2]. For stage III colorectal cancer, oxaliplatin plus fluoropyrimidine combination regimens have been confirmed as effective by the randomized controlled trials (RCT) [3–6], although they were not established at the start of this trial. In contrast, no effective adjuvant regimen has been established for CLM [7,8], which is classified as stage IV in simultaneous occurrence [9,10].

In general, patients with stage III disease can tolerate adjuvant regimens, such as oxaliplatin with folinic acid and 5-fluorouracil (FOLFOX) or capecitabine. However, it would be difficult for patients undergoing hepatic resection to tolerate these regimens, because hepatic resection is more invasive than colorectal resection. Indeed, the completion rates of the adjuvant chemotherapy (5-fluorouracil with folinic acid) after hepatic resection [9] were lower than that of the same regimen after colorectal resection [3]. Thus, a safe and effective adjuvant regimen with sufficient adherence has been required for treatment of CLM.

Uracil-tegafur (UFT) is an oral 5-fluorouracil preparation combining tegafur and uracil in a molar ratio of 1:4. Tegafur is metabolized to 5-fluorouracil in the liver, and uracil competitively inhibits the main metabolizing enzyme of 5-fluorouracil, thereby increasing serum concentrations of 5-fluorouracil. To date, UFT combined with an oral folinic acid preparation (leucovorin; LV) has been used as one of the standard adjuvant regimens for stage III colon cancer [11,12]. Because UFT/LV can be administered orally and conveniently, it may have practical advantages in treating patients after hepatic resection as suggested by the previous trials [13,14]. Thus, we conducted a RCT to test the hypothesis that UFT/LV regimen would more effectively prevent recurrence after resection of CLM than surgery alone.

Because we consider the tolerability and safety of adjuvant chemotherapy as important factors in selecting an appropriate treatment, we have previously shown the results concerning the safety of the UFT/LV regimen for CLM in the first report [15]. In this second report, we show the main results to evaluate the efficacy of the UFT/LV in preventing recurrence.

## Patients and Methods

### Study Design

This multicenter, open-label, RCT was conducted at 5 university hospitals, 4 regional medical centers, and 2 cancer centers in Japan. The trial was conducted in accordance with the Declaration of Helsinki and the ethical principles for clinical studies in Japan. The protocol and its revision were firstly approved by “The Research Ethics Committee of the Faculty of Medicine and Graduate School of Medicine of the University of Tokyo” (No. P2003022-11X), which were also approved in each participating center. The full names of all the institutional review board (IRB) are as follows; the IRB of Cancer Institute Hospital, the IRB of Nihon University School of Medicine, the IRB of Shinshu University School of Medicine, the IRB of National Defense Medical College, the IRB of JCHO Tokyo Yamate Medical Center, the IRB of Showa General Hospital, the IRB of Ibaraki Prefectural Central Hospital and Cancer Center, the IRB of Juntendo University School of Medicine, and the IRB of Japanese Red Cross Medical Center. An English summary of the protocol has been disclosed at http://www.umin.ac.jp/ctr/index.htm (UMIN Clinical Trials Registry; C000000013). All patients provided written informed consent.

### Patients

The trial design and safety outcomes have been reported previously [15]. In brief, patients with 20 to 80 years of age undergoing curative hepatic resection for CLM were eligible, if they had adequate organ functions defined as the following serum laboratory values: white blood cell count 4,000–12,000/μL, platelet count ≥100,000/μL, hemoglobin ≥9.0g/dL, total bilirubin ≤1.5mg/dL, alanine aminotransferase ≤100IU/L, creatinine ≤1.5mg/dL, and albumin ≥3.0g/dL. In this trial, tumor surface exposure without injury of tumor was regarded as macroscopically curative but R1 resection. A patient receiving chemotherapy before detection of CLM (e.g., adjuvant after surgery for the primary colorectal disease) could be included, if at least 3 months had passed after the last drug administration.

Exclusion criteria were extrahepatic metastasis; other previous or concurrent malignant disorders; history of local or systemic chemotherapy or radiotherapy for CLM; postoperative dysfunction of any organ; poorly controlled diabetes mellitus or hypertension; history of myocardial infarction within past 6 months or unstable angina; liver cirrhosis; or interstitial pneumonia, pulmonary fibrosis, or pulmonary emphysema. Patients treated with insulin were also regarded as those with poorly controlled diabetes mellitus and were excluded. To exclude lung and minute liver metastasis, preoperative plain X-ray or lung computed tomography and intraoperative ultrasonography were performed in all patients.

### Treatments

After hepatic resection for CLM, patients were randomly assigned in a 1:1 ratio to receive oral UFT/LV or surgery alone by the stochastic minimization method with a random element using the 5 factors: institution, timing of development of CLM (synchronous [defined by disease-free interval shorter than 12 months] or metachronous), number of CLM (single or multiple), location of the primary carcinoma (colon or rectum), and timing of hepatic resection (first or second). In the randomization process, first, the investigator in charge of this RCT of each institution accessed the assignment system via internet managed by the third party (the University Hospital Medical Information Network). Second, the person of this third party performed assignment, and sent its result to the investigator.

In June 2005, we revised the protocol to increase recruitment rate. After the revision, patients with a second intrahepatic recurrence after hepatic resection for initial CLM became eligible, and the timing of hepatic resection was added to the stratification factors.

In patients of the UFT/LV group, 5 cycles of UFT/LV (UFT 300mg/m^2^ of body-surface area and LV 75mg/day for 28 days followed by a 7-day rest per cycle) were started within 8 weeks after surgery. Protocol treatment with UFT/LV was discontinued in the following conditions: recurrence; the treatment could not be resumed for more than 15 days; the dose had to be reduced by more than one level; the patient wished to discontinue the treatment; the investigator considered it difficult to continue the treatment; or other reasons, as previously described [5].

### Outcomes

After randomization, patients in both groups underwent ultrasonography every 3 months, enhanced computed tomography every 6 months, and blood sampling to measure tumor markers (carcinoembryonic antigen and carbohydrate antigen 19–9) every month for up to 1 year after hepatic resection. After 1 year, the frequencies of ultrasonography and blood sampling were decreased to once every 6 and 2 months, respectively.

The primary endpoint was recurrence-free survival (RFS). The secondary endpoints included overall survival (OS) and safety. RFS was defined as the interval between the date of randomization and the date of diagnosis of the first recurrence, death, or the last follow-up visit. Recurrence or death from colorectal carcinoma, whichever occurs the earliest, shall be counted as the event, whereas death from other diseases without recurrence shall be as the censor. Recurrence was defined as reappearance of a lesion with typical findings on predefined standard imaging modalities (enhanced computed tomography, ultrasonography, bone scintigraphy, positron emission tomography, or a combination thereof). When recurrence or another malignancy developed, UFT/LV treatment was withdrawn. Patients with localized recurrence in the liver underwent repeated resection if their liver function remained adequate and curative surgery was possible. In patients with lung metastasis, surgical resection was considered if the number of metastases was 3 or fewer. Other patients received systemic chemotherapy.

### Statistical Analyses

We hypothesised that UFT/LV treatment would improve the 3-year RFS rate from 20% (based on our unpublished data) to 35%. Under the assumption that the registration would be completed within 3 years and the registered patients would be followed up for total 6 years, we estimated that 180 patients would be required to detect this difference with a type I error level of 5% (2-sided) and a power of 75%. At start of the registration, the recruitment period was set as 3 years. Analyses for the primary endpoint were scheduled after follow-up period of 3 years without plan of interim analysis.

Patients who violated the eligibility criteria were excluded from the efficacy analysis, whereas patients who did not receive the assigned treatment were excluded from the previous safety analysis [15] The institution of the corresponding author collected the data from the participating institutions, which were analyzed by the statistician (Y.M.) under masking. The survival curves of the treatment groups were calculated by the Kaplan-Meier method and were compared by the stratified log-rank test. Under the proportional hazards assumption, the effects of UFT/LV on RFS or OS were calculated as stratified hazard ratios with 95% confidence intervals, which were adjusted by the 4 stratification factors (timing of development of CLM, number of CLM, location of the primary carcinoma, and timing of hepatic resection). Statistical significance level was defined as P<0.05 (2-sided). Additionally, RFS rates were compared between the treatment groups in the subgroups of patients with single or multiple metastases and the subgroups of patients with synchronous or metachronous metastases. Furthermore, the locations and resection rates were compared between the treatment groups with the Mantel trend test and Fisher’s exact test, respectively. All analyses were performed with SAS^®^ computer software version 9.3 (SAS Institute Inc., Cary, NC, USA).

### Access to Study Data

All authors had access to the study data and approve the final version of the manuscript.

## Results

**Fig 1:**
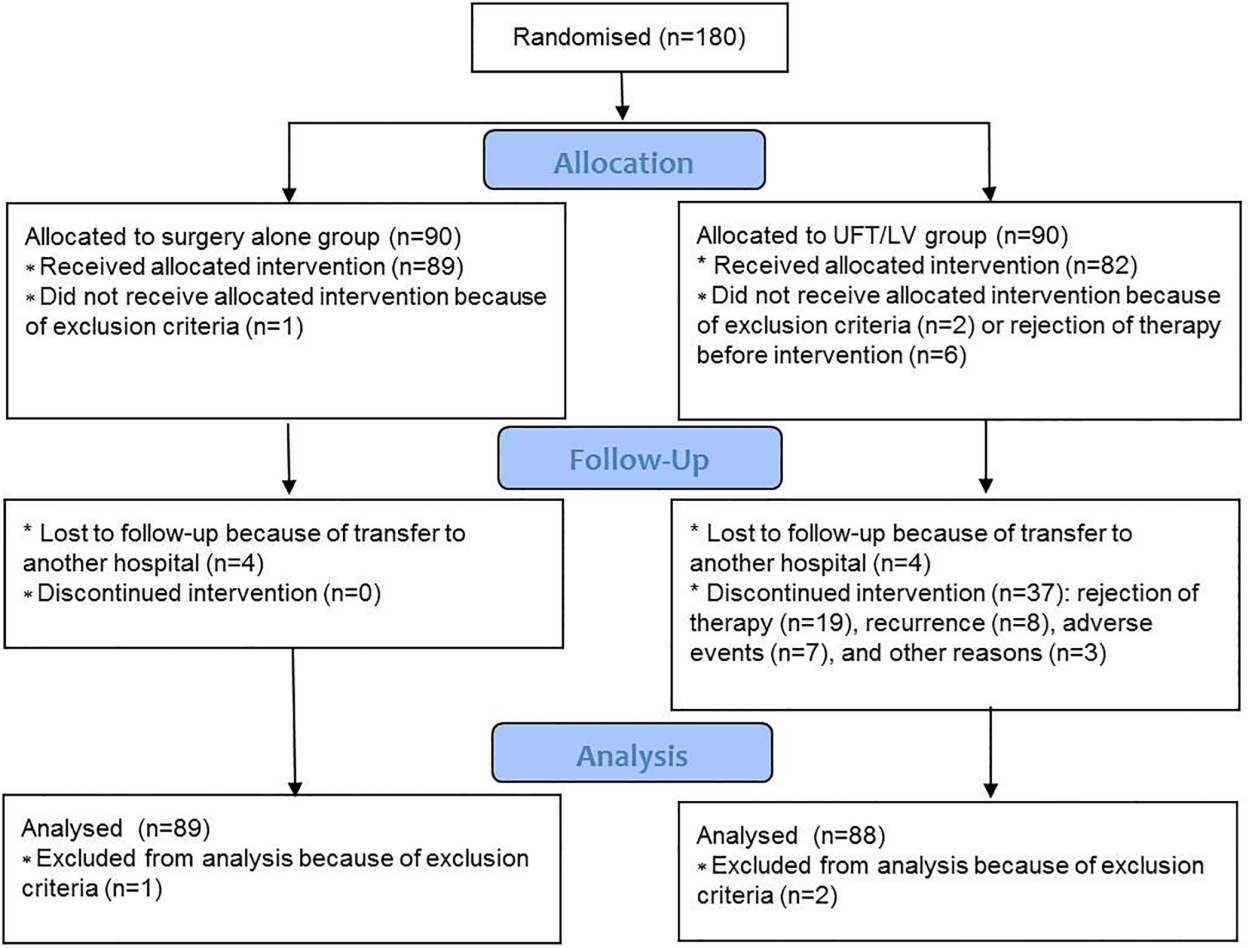
The trial profile. The Participant flow is shown.

From 2/2/2004 to 28/12/2010, 180 patients were assigned to the UFT/LV (n = 90) or surgery alone (n = 90) groups (Fig 1). Of these, 3 patients were excluded from the efficacy analysis because they violated the eligibility criteria (2 with poorly controlled diabetes mellitus in the UFT/LV group and 1 in the surgery alone who received UFT/LV before randomization). The remaining 177 patients were included in the analysis. After the scheduled follow-up period of 3 years, we collected data concerning prognosis of the registered patients at 28/12/2013, which were fixed for the analyses.

In the UFT/LV group, 6 patients did not receive the treatment because of rejection before intervention, and 37 patients (42%) discontinued the treatment according to the protocol because of rejection during intervention (n = 19), recurrence before (n = 2) and during (n = 6) intervention, adverse events (n = 7), or other reasons (n = 3). All adverse events resolved by conservative therapy. No chemotherapy-related death occurred. The baseline characteristics were similar between the treatment groups, except for lymph node status of the primary diseases (Table 1).

**Table 1:** Baseline characteristics.

	Surgery alone	UFT/LV	P-value
	n = 89	n = 88	
Age (years), mean (SD)	64.4 (9.2)	62.3 (8.5)	0.119
Gender, n (%)			0.424
Male	63 (70.8)	57 (64.8)	
Female	26 (29.2)	31 (35.2)	
Primary disease			
Location, n (%)			0.441
Colon	58 (65.2)	52 (59.1)	
Rectum	31 (34.8)	36 (40.9)	
Lymph-node metastasis, n (%)*			0.041
n0	29 (33.0)	41 (48.2)	
n+	59 (67.0)	44 (51.8)	
Liver metastasis			
Maximum tumor size (mm), mean (SD)	38.73 (25.95)	39.98 (29.78)	0.767
Maximum tumor size (mm), n (%)†			0.597
≤30	48 (53.9)	45 (51.1)	
>30 to ≤50	23 (25.8)	22 (25.0)	
>50	18 (20.2)	21 (23.9)	
Tumor number, mean (SD)	2.8 (2.8)	3.2 (3.9)	0.363
Tumor number, n (%)			0.367
Single	44 (49.4)	37 (42.0)	
Multiple	45 (50.6)	51 (58.0)	
Synchronous or metachronous, n (%)			1.000
Synchronous	40 (44.9)	39 (44.3)	
Metachronous	49 (55.1)	49 (55.7)	
Hepatectomy, n (%)			0.444
First	84 (94.4)	86 (97.7)	
Second	5 (5.6)	2 (2.3)	
Surgical margin (mm), mean (SD)	6.1 (7.2)	7.3 (14.1)	0.486
Tumor differentiation, n (%)			0.838
Well	32 (36.0)	31 (35.2)	
Moderate	55 (61.8)	56 (63.6)	
Poor	2 (2.2)	1 (1.1)	
Time from liver operation to	27.0 (14, 77)	28.5 (14, 55)	0.794
randomisation ‡(days), median (min, max)			

Fisher's exact test / t-test unless otherwise specified;

*Data were missing in 1 and 3 patients of the surgery alone and UFT/LV groups, respectively.

^†^ Mantel trend test;

^‡^ Wilcoxon rank sum test

**Fig 2:**
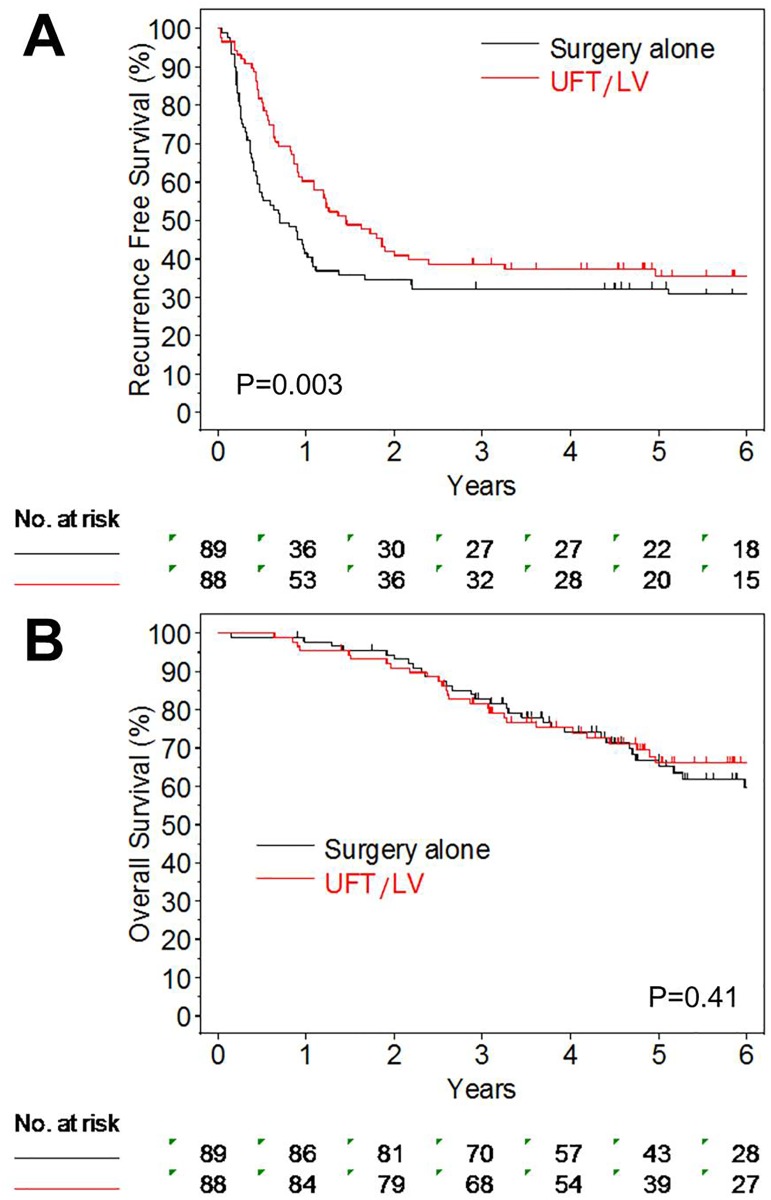
Results of analyses of the primary and secondary endpoints. A: The recurrence-free survival curves of the UFT/LV group (red line) and surgery alone group (black line) group are shown. The 3-year recurrence-free rate was significantly higher in the UFT/LV group than in the surgery alone group (38.6% vs. 32.3%, P = 0.003). B: The overall survival curves of the two groups are shown. The 5-year overall survival rates of the two groups were similar (66.1% vs. 66.8%, P = 0.409).

The median follow-up was 4.76 (range, 0.15–9.84) years, which was calculated for the whole analyzed patients including survivors without recurrence. The RFS at 3 years was higher in the UFT/LV group (38.6%; 95% confidence interval, 28.5%-48.6%) than in the surgery alone (32.3%; 95% confidence interval, 22.8%-42.1%; Fig 2-A). The median RFS (95% confidence interval) in the UFT/LV and surgery alone groups were 1.45 years (0.96–2.16) and 0.70 years (0.44–1.07), respectively. UFT/LV significantly prolonged the RFS compared with surgery alone with the hazard ratio of 0.56 (95% confidence interval, 0.38–0.83; P = 0.003). The OS rates at 5 years were similar between the UFT/LV and surgery alone groups (66.1% vs. 66.8%, Fig 2-B) with the hazard ratio of 0.80 (95% confidence interval, 0.48–1.35; P = 0.409). The median OS could not be calculated because of insufficient number of events.

**Fig 3:**
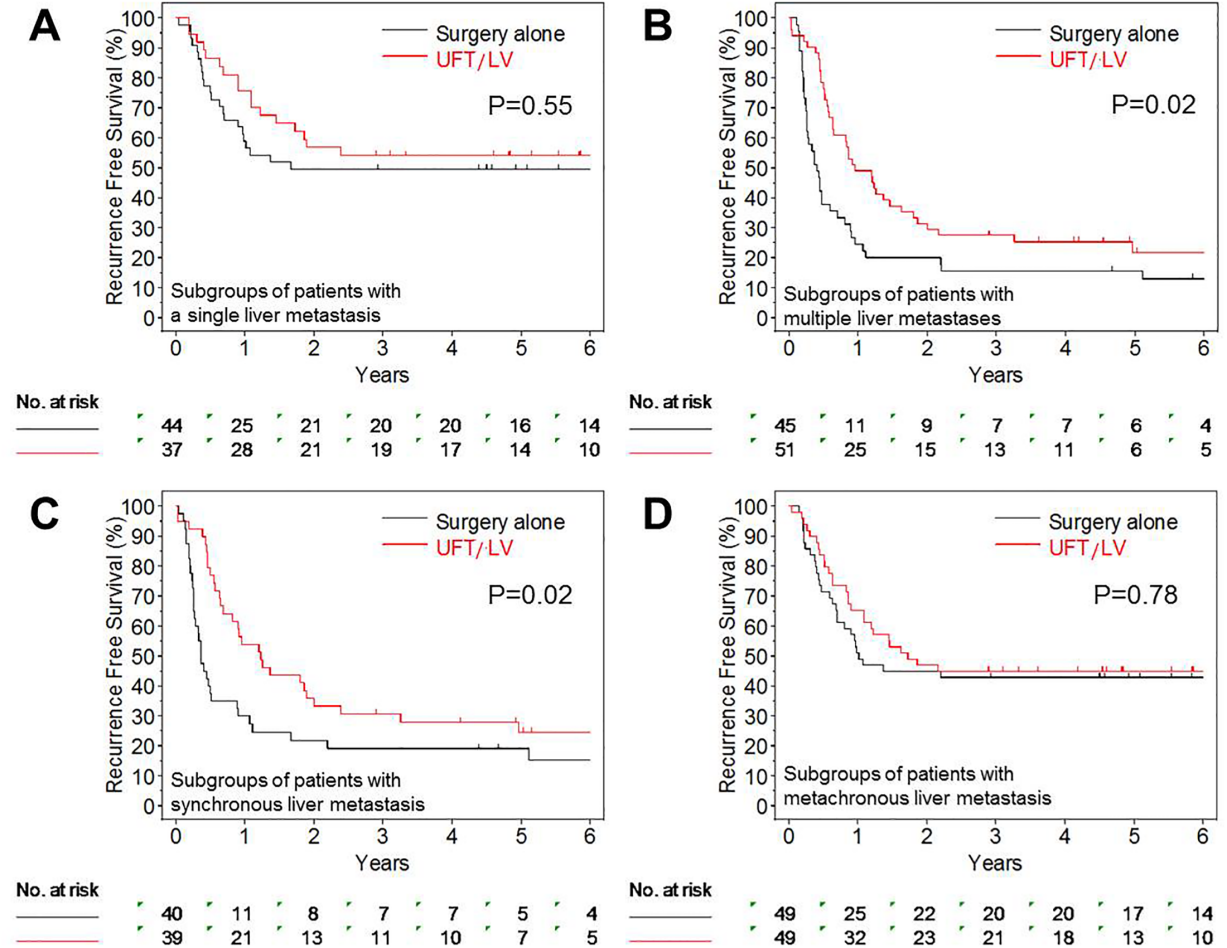
Results of subgroup analyses. A: The recurrence-free survival curves of the UFT/LV group (red line) and surgery alone group (black line) are shown for patients with a single liver metastasis. B: The recurrence-free survival curves of the two groups are shown for patients with multiple liver metastases. C: The recurrence-free survival curves of the two groups are shown for patients with synchronous liver metastases. D: The recurrence-free survival curves of the two groups are shown for patients with metachronous liver metastases.

In the subgroup of patients with multiple tumors, the RFS was higher in the UFT/LV group than in the surgery alone (P = 0.019, Fig 3-B), despite similar RFS in patients with single tumors (P = 0.554, Fig 3-A). In the subgroup of patients with synchronous CLM, the RFS was higher in the UFT/LV group than in the surgery alone (P = 0.023, Fig 3-C), despite similar RFS in the subgroup of patients with metachronous CLM (P = 0.782, Fig 3-D).

During the follow-up, 59 (68.5%) patients in the UFT/LV group and 61 (69.3%) in the surgery alone had recurrence. The locations and treatments of the first recurrences are shown in Table 2. The remnant liver was the main site of recurrence in both the UFT/LV (40.7%) and surgery alone (34.4%) groups. The resection rates for the first recurrence were similar between the UFT/LV and surgery alone groups (55.9% vs. 41.0%).

**Table 2:** Locations and treatments of first recurrence.

	Surgery alone	UFT/LV	P value
	n = 61	n = 59	
Location, n (%)†			0.514
Intrahepatic only	21 (34.4)	24 (40.7)	
Intrapulmonary only	10 (16.4)	13 (22.0)	
Both in the liver and lung	8 (13.1)	4 (6.8)	
Extrahepatic and extrapulmonary	22 (36.1)	18 (30.5)	
Treatment performed, n (%)††			0.101
Non-surgical treatments	36 (59.0)	26 (44.1)	
Resection	25 (41.0)	33 (55.9)	

^†^ Mantel trend test;

^††^ Fisher's exact test

Because the distributions of lymph node status of the primary colorectal disease were different between the two groups (Table 1), we additionally calculated a hazard ratio of use of UFT/LV against surgery alone for RFS. In this analysis, total 4 cases with unknown lymph node status were deleted, and Cox’s proportional hazard model was used with adjustment of lymph node status. The hazard ratio was 0.67 (95% confidence interval; 0.46–0.99, P = 0.044).

## Discussion

In this study, adjuvant UFT/LV therapy significantly reduced recurrence after hepatic resection for CLM compared with surgery alone with the hazard ratio of 0.56 (P = 0.0003), which indicates UFT/LV as the potential candidate of standard treatment in this setting.

In the previous RCT, intravenous administration of 5-fluorouracil plus folinic acid might prevent recurrence [9], although this study failed to confirm the efficacy on OS [16]. Given the advantage of UFT/LV as an oral preparation of 5-fluorouracil plus folinic acid, we believe that UFT/LV is an appropriate and equally potent treatment option in preventing recurrence after hepatic resection for CLM. In addition, UFT/LV was more effective in the subgroups of patients with multiple and synchronous metastases. Because multiplicity and the synchronous development are associated with higher risks of recurrence than single and metachronous development, respectively, our results suggest that UFT/LV may be more effective for more advanced disease. However, this must be confirmed by further investigations.

The results of four RCTs about adjuvant therapies were published [17–20], however, no study could confirm positive impacts on long-term outcomes, except for the above study [9,16], which indicated survival benefits only on RFS not but OS. In addition to them, our results indicated again positive effects of UFT/LV to prevent recurrence after resection of CLM, which would be clinically meaningful in the current practices, where few effective regimens are available. Strictly speaking, the significance of addition of oxaliplatin to 5-fluorouracil plus folinic acid would also remain unclear for colorectal metastases in our opinion. If practically possible, it is reasonable to conduct a next RCT to evaluate the significance of oxaliplatin to UFT/LV after the completion of this RCT.

Perioperative chemotherapy seems to be regarded as the standard management of CLM, based on the previous RCT on perioperative FOLFOX4 [10], especially in the Western centers. However, the conclusion of this study might be weak as other investigator has pointed out [21], because the intention-to-treat analysis failed to show efficacy in preventing recurrence [10] and further follow-up did not find significant survival benefit of FOLFOX4 on OS [22]. Failure of the above well-designed, appropriately powered study to show significant improvement in OS may indicate the innate difficulty to conduct RCTs in this setting. In a recent retrospective study, Araujo suggested that postoperative adjuvant chemotherapy would have similar effects compared to perioperative one [23]. Although it is impossible to evaluate the significance of perioperative therapy by our results, we think that postoperative UFT/LV chemotherapy is sufficiently useful as well as perioperative FOLFOX4. In addition, considering that even FOLFOX, which is a more toxic regimen than UFT/LV, exhibits only RFS benefit but not OS benefit, this fact would indirectly imply the potential advantages of adjuvant UFT/LV.

The second possible advantage of adjuvant UFT/LV is the higher resection rate than perioperative chemotherapy, because resection is often precluded by deterioration of tumor factors and/or liver function during preoperative chemotherapy. Although our results do not directly support the superiority of postoperative chemotherapy over perioperative, our results suggest that postoperative UFT/LV can be positioned at least as an alternative treatment to perioperative chemotherapy. Of course, a well-designed RCT is required to confirm our claim.

The third advantage of UFT/LV is its safety with acceptable adherence, which was shown in our previous report [15]. No mortality related to UFT/LV was observed in this study with the acceptable treatment completion rate (54.9%). The incidence of grade 3 or 4 adverse events was 12.2%, which were resolved by conservative treatments. Although concern has been expressed that the risk of chemotherapy after hepatic resection might be higher than that after surgery for primary colorectal carcinoma [9,20], our results indicate this is not the case.

The most important question raised by our results is why the OS rates were similar in the treatment groups, despite the significant difference in the RFS. As suggested by a recent RCT [24], we were concerned that the addition of UFT/LV might deteriorate tumor status. To address this question, we investigated the types of first recurrences and treatments for them. As Table 2 shows, there was no difference in the locations or the treatments of the first recurrences between the two groups. Although the currently available data are immature and further follow-up is needed, our results suggest that the UFT/LV regimen had no negative influence on the type of recurrence. It would be another possible explanation that the UFT/LV regimen cannot prevent recurrence itself, but only delay a timing of recurrence. Even if it is true, however, we believe that delay of recurrence would be practically valuable for a patient struggling against CLM.

The 5-year OS rates were as high enough (over 66%) in both the surgery alone and UFT/LV groups, compared to those of other previous reports ranging 39.6% to 52.8% [1,2,16,22]. Potential differences in OS between the treatment groups were thus estimated to be quite small and undetectable in practically executable clinical trials. Even if patients with more advanced tumor status are planned to be included in the future study, it would most likely be difficult to detect differences in OS between the two groups.

Our study had several limitations. First, the follow-up period was not long enough, and the numbers of deaths were relatively low in both groups. On longer follow-up period, the significant difference in RFS between the treatment groups shown in the current analyses might lead to significant difference in OS. To confirm this assumption, we are planning to perform secondary analyses, focusing on OS with follow-up of 2 years longer (i.e., 5 years after the completion of enrollment). As a possible reason for the similar OS in this study, we assume that second-line and subsequent treatments, such as repeated resection for liver or lung metastases (or both) might be effective enough ([25] to minimize the effects of the initial therapy, but this remains speculative and must await the results of future analyses.

Second, the higher association of lymph node metastasis of the primary disease in the surgery alone group might affect the results, because it would have negative impacts on prognosis. However, the results of the additional analysis (hazard ratio of UFT/LV; 0.67, 95% confidence interval; 0.46–0.99, P = 0.044) indicate that UFT/LV would be also effective to prevent recurrence, as was the same with the main results of this study.

Third, the recruitment period was long up to nearly 7 years, which was also previously reported [9], possibly because of the study design, using surgery alone as a control. Candidate patients and their families might hesitate to participate in this trial, because they were apprehensive about the possibility of being assigned to the surgery alone [26]. In fact, we acknowledge that low recruitment rate might have affected our conclusion. However, we believe that this study provided important findings because it would be impossible to conduct another RCT with surgery alone as a control arm in patients with CLM.

Forth, 75% as the detection power was slightly lower than the standard (80%). The reason for this was to set the targeted number of patients within practically possible range. However, this limitation would be a minor problem, because the significant difference was found for the primary endpoint as a result.

Another limitation would be minor spread of UFT in the Western countries, which is a practical but minor problem. The most important point of our results is the efficacy of oral anticancer medicine as an adjuvant for CLM, which can be substituted by other oral medicines available in the Western, such as Capecitabine and S-1. Now that the use of molecular-targeted drugs for CLM is not promising suggested by the new EPOC trial (24), the role of the common oral regimens should be reappraised, especially for a patient who does not want to undergo strong adjuvant chemotherapy after hepatic resection.

In conclusion, oral UFT/LV adjuvant chemotherapy is an effective and safe regimen that can be recommended as an alternative choice after hepatic resection for CLM.

## Supporting Information

S1 File CONSORT checklist. (DOC)

S2 File Clinical study protocol. (PDF)

S3 File List of Modifications of the Protocol. (PDF)

S4 File Data set. (SAS7BDAT)

## References

[1] Abdalla EK, Vauthey JN, Ellis LM, Ellis V, Pollock R, Broglio KR, et al Recurrence and outcomes following hepatic resection, radiofrequency ablation, and combined resection/ablation for colorectal liver metastases. Ann Surg 2004;239:818–25. 15166961 10.1097/01.sla.0000128305.90650.71 PMC1356290

[2] Beppu T, Sakamoto Y, Hasegawa K, Honda G, Tanaka K, Kotera Y, et al A nomogram predicting disease-free survival in patients with colorectal liver metastases treated with hepatic resection: multicenter data collection as a project study for hepatic surgery of the Japanese Society of Hepato-Biliary-Pancreatic Surgery. J Hepatobiliary Pancreat Sci 2012;19:72–84. 10.1007/s00534-011-0460-z 22020927

[3] André T, Boni C, Mounedji-Boudiaf L, Navarro M, Tabernero J, Hickish T, et al Multicenter International Study of Oxaliplatin/5-Fluorouracil/Leucovorin in the Adjuvant Treatment of Colon Cancer (MOSAIC) Investigators. Oxaliplatin, fluorouracil, and leucovorin as adjuvant treatment for colon cancer. N Engl J Med 2004;350:2343–51. 15175436 10.1056/NEJMoa032709

[4] Kuebler JP, Wieand HS, O'Connell MJ, Smith RE, Colangelo LH, Yothers G, et al Oxaliplatin combined with weekly bolus fluorouracil and leucovorin as surgical adjuvant chemotherapy for stage II and III colon cancer: results from NSABP C-07. J Clin Oncol 2007;25:2198–204. 17470851 10.1200/JCO.2006.08.2974

[5] André T, Boni C, Navarro M, Tabernero J, Hickish T, Topham C, et al Improved overall survival with oxaliplatin, fluorouracil, and leucovorin as adjuvant treatment in stage II or III colon cancer in the MOSAIC trial. J Clin Oncol 2009;27:3109–16. 10.1200/JCO.2008.20.6771 19451431

[6] Yothers G, O'Connell MJ, Allegra CJ, Kuebler JP, Colangelo LH, Petrelli NJ, et al Oxaliplatin as adjuvant therapy for colon cancer: updated results of NSABP C-07 trial, including survival and subset analyses. J Clin Oncol 2011;29:3768–74. 10.1200/JCO.2011.36.4539 21859995 PMC3188282

[7] Choti M, Pawlick TM. Neoadjuvant or adjuvant therapy for patients with resectable liver metastases. Curr Colorectal Cancer Rep 2008; 4: 160–166

[8] Arnold D, Stein A. Adjuvant therapy after liver resection for colorectal cancer metastasis: What is the evidence? Curr Colorectal Cancer Rep 2011; 7: 180–186

[9] Portier G, Elias D, Bouche O, Rougier P, Bosset JF, Saric J, et al Multicenter randomized trial of adjuvant fluorouracil and folinic acid compared with surgery alone after resection of colorectal liver metastases: FFCD ACHBTH AURC 9002 trial. J Clin Oncol 2006;24:4976–82. 17075115 10.1200/JCO.2006.06.8353

[10] Nordlinger B, Sorbye H, Glimelius B, Poston GJ, Schlag PM, Rougier P, et al EORTC Gastro-Intestinal Tract Cancer Group; Cancer Research UK; Arbeitsgruppe Lebermetastasen und-tumoren in der Chirurgischen Arbeitsgemeinschaft Onkologie (ALM-CAO); Australasian Gastro-Intestinal Trials Group (AGITG); Fédération Francophone de Cancérologie Digestive (FFCD). Perioperative chemotherapy with FOLFOX4 and surgery versus surgery alone for resectable liver metastases from colorectal cancer (EORTC Intergroup trial 40983): a randomised controlled trial. Lancet 2008;371:1007–16. 18358928 10.1016/S0140-6736(08)60455-9 PMC2277487

[11] Lembersky BC, Wieand HS, Petrelli NJ, O'Connell MJ, Colangelo LH, Smith RE, et al Oral uracil and tegafur plus leucovorin compared with intravenous fluorouracil and leucovorin in stage II and III carcinoma of the colon: results from National Surgical Adjuvant Breast and Bowel Project Protocol C-06. J Clin Oncol 2006;24:2059–64. 16648506 10.1200/JCO.2005.04.7498

[12] Shimada Y, Hamaguchi T, Mizusawa J, Saito N, Kanemitsu Y, Takiguchi N, et al Randomised phase III trial of adjuvant chemotherapy with oral uracil and tegafur plus leucovorin versus intravenous fluorouracil and levofolinate in patients with stage III colorectal cancer who have undergone Japanese D2/D3 lymph node dissection:final results of JCOG0205. Eur J Cancer 2014;50:2231–40. 10.1016/j.ejca.2014.05.025 24958736

[13] Douillard JY, Hoff PM, Skillings JR, Eisenberg P, Davidson N, Harper P, et al Multicenter phase III study of uracil/tegafur and oral leucovorin versus fluorouracil and leucovorin in patients with previously untreated metastatic colorectal cancer. J Clin Oncol 2002;20:3605–16. 12202661 10.1200/JCO.2002.04.123

[14] Borner MM, Schoffski P, de Wit R, Caponigro F, Comella G, Sulkes A, et al Patient preference and pharmacokinetics of oral modulated UFT versus intravenous fluorouracil and leucovorin: a randomised crossover trial in advanced colorectal cancer. Eur J Cancer 2002;38:349–58. 11818199 10.1016/s0959-8049(01)00371-9

[15] Saiura A, Yamamoto J, Hasegawa K, Oba M, Takayama T, Miyagawa S, et al A combination of oral uracil-tegafur plus leucovorin (UFT+LV) is a safe regimen for adjuvant chemotherapy after hepatectomy in patients with colorectal cancer: Safety report of the UFT/LV study. Drug Discov Ther 2014;8:48–56. 24647158 10.5582/ddt.8.48

[16] Mitry E, Fields AL, Bleiberg H, Labianca R, Portier G, Tu D, et al Adjuvant chemotherapy after potentially curative resection of metastases from colorectal cancer: a pooled analysis of two randomized trials. J Clin Oncol 2008;26:4906–11. 10.1200/JCO.2008.17.3781 18794541

[17] Lorenz M, Müller HH, Schramm H, Gassel HJ, Rau HG, Ridwelski K, et al Randomized trial of surgery versus surgery followed by adjuvant hepatic arterial infusion with 5-fluorouracil and folinic acid for liver metastases of colorectal cancer. German Cooperative on Liver Metastases (Arbeitsgruppe Lebermetastasen). Ann Surg 1998;228:756–62. 9860474 10.1097/00000658-199812000-00006 PMC1191593

[18] Rudroff C, Altendorf-Hoffmann A, Stangl R, Scheele J. Prospective randomised trial on adjuvant hepatic-artery infusion chemotherapy after R0 resection of colorectal liver metastases. Langenbecks Arch Surg 1999;384:243–9 10437612 10.1007/s004230050199

[19] Schulze T, Kemmner W, Weitz J, Wernecke KD, Schirrmacher V, Schlag PM. Efficiency of adjuvant active specific immunization with Newcastle disease virus modified tumor cells in colorectal cancer patients following resection of liver metastases: results of a prospective randomized trial. Cancer Immunol Immunother 2009;58:61–9. 10.1007/s00262-008-0526-1 18488223 PMC11030620

[20] Ychou M, Hohenberger W, Thezenas S, Navarro M, Maurel J, Bokemeyer C, et al A randomized phase III study comparing adjuvant 5-fluorouracil/folinic acid with FOLFIRI in patients following complete resection of liver metastases from colorectal cancer. Ann Oncol 2009;20:1964–70. 10.1093/annonc/mdp236 19567451

[21] Fong Y. Chemotherapy and resection for colorectal metastases. Lancet Oncol 2013;14:1148–9. 10.1016/S1470-2045(13)70478-9 24120479

[22] Nordlinger B, Sorbye H, Glimelius B, Poston GJ, Schlag PM, Rougier P, et al EORTC Gastro-Intestinal Tract Cancer Group; Cancer Research UK; Arbeitsgruppe Lebermetastasen und–tumoren in der Chirurgischen Arbeitsgemeinschaft Onkologie (ALM-CAO); Australasian Gastro-Intestinal Trials Group (AGITG); Fédération Francophone de Cancérologie Digestive (FFCD). Perioperative FOLFOX4 chemotherapy and surgery versus surgery alone for resectable liver metastases from colorectal cancer (EORTC 40983): long-term results of a randomised, controlled, phase 3 trial. Lancet Oncol 2013;14:1208–15. 24120480 10.1016/S1470-2045(13)70447-9

[23] Araujo R, Gonen M, Allen P, Blumgart L, DeMatteo R, Fong Y, et al Comparison between perioperative and postoperative chemotherapy after potentially curative hepatic resection for metastatic colorectal cancer. Ann Surg Oncol 2013;20:4312–21. 10.1245/s10434-013-3162-8 23897009

[24] Primrose J, Falk S, Finch-Jones M, Valle J, O'Reilly D, Siriwardena A, et al Systemic chemotherapy with or without cetuximab in patients with resectable colorectal liver metastasis: the New EPOC randomised controlled trial. Lancet Oncol 2014;15:601–11. 10.1016/S1470-2045(14)70105-6 24717919

[25] Oba M, Hasegawa K, Matsuyama Y, Shindoh J, Mise Y, Aoki T, et al Discrepancy between recurrence-free survival and overall survival in patients with resectable colorectal liver metastases: a potential surrogate endpoint for time to surgical failure. Ann Surg Oncol 2014;21:1817–24. 10.1245/s10434-014-3504-1 24499828

[26] Kokudo N, Hasegawa K, Makuuchi M. Control arm for surgery alone is needed but difficult to obtain in randomized trials for adjuvant chemotherapy after liver resection for colorectal metastases. J Clin Oncol 2007;25:1299–300. 17401028 10.1200/JCO.2006.09.9069

