# Supplementary material for: Adjuvant Oral Uracil-Tegafur with Leucovorin for Colorectal Cancer Liver Metastases: A Randomized Controlled Trial
Source: PLoS One. 2016 Sep 2;11(9):e0162400. doi: 10.1371/journal.pone.0162400 (PMC5010179; doi:10.1371/journal.pone.0162400)
Supplement: S2 File — (PDF) [file pone.0162400.s002.pdf]

Clinical Study Protocol：

A randomized controlled trial for adjuvant LV+UFT therapy after resection of liver metastasis from colorectal carcinoma.

Principal Investigator: Norihiro Kokudo
Department of Surgery, Graduate School of Medicine, University of Tokyo
Telephone +81-3-5800-8654
Fax +81-3-5800-8844
E-mail KOKUDO-2SU@h.u-tokyo.ac.jp
Urgent contact In-hospital PHS: 30350

Preparation date	version
November 4, 2003	1
March 25, 2004	2
April 14, 2005	3
April 25, 2006	4
April 3, 2008	5
May 21, 2009	6

0. Summary

0.1. Study design

A randomized open-label clinical study involving parallel group comparison with untreated controls and an explorative clinical study

0.2. Objectives

The study is aimed at evaluating the usefulness of postoperative adjuvant chemotherapy in colorectal cancer patients with liver metastasis who have undergone macroscopically curative resection of the liver metastasis, through comparing the endpoints (recurrence-free survival (residual liver recurrence and/or extrahepatic recurrence), overall survival) between patients treated by hepatectomy alone ("the surgery alone group") and those treated by hepatectomy and tegafur-uracil [UFT] + leucovorin [LV] (UFT/LV) therapy.

Primary endpoint: Recurrence-free survival

Secondary endpoints: Overall survival, safety

0.3. Inclusion criteria

Prospective subjects meeting the following criteria will be considered eligible for this study.

1) Patients with histopathologically proven colorectal cancer with liver metastasis who have undergone surgical resection for foci of liver metastasis

2) Among the patients who have undergone macroscopically curative resection for foci of liver metastasis

[1] Cases confirmed by intraoperative ultrasonography to be free of residual tumor of liver metastasis

[2] No tumor invasion of the surgical cut surface (zero (0) mm is acceptable)

[3] Cases confirmed by palpation of hilar and paraaortic lymph nodes with no metastasis. Cases with evident metastasis in these lymph nodes will be excluded. (Sampled for histological examination as needed)

3) Patients undergoing treatment of liver metastasis for the first time or who have undergone hepatectomy once before. The liver metastasis may be synchronous or metachronous, but the period from resection of the primary tumor to diagnosis of the liver metastasis may not exceed 12 months. Patients undergoing rehepatectomy shall be eligible only if they were not registered for this study at the time of the first hepatectomy.

*There is no restriction depending on whether the first hepatectomy was carried out at own study institution or at another institution, the period from the first resection of liver metastasis to the rehepatectomy, or the presence/absence of adjuvant chemotherapy. However, Criterion 5) of Section 0.3. needs to be satisfied.

4) No extrahepatic lesions

[1] Confirmed by plain chest X-ray or CT to have no lung metastasis.

[2] In cases of metachronous liver metastasis, confirmed by Barium enema or colonoscopy (or pelvic CT) to have no evidence of local recurrence.

[3] Confirmed by upper abdominal CT to have no enlarged lymph nodes in the hepatic hilum or the paraaortic region.

5) No previous history of treatment for hepatic metastasis (e.g., local/systemic chemotherapy or radiotherapy)

   There is no restriction on chemotherapy received before detection of the first liver metastasis (e.g., adjuvant chemotherapy after surgery for colorectal cancer), but at least 3 months should have passed after the last drug dose of such chemotherapy.

6) Age between 20 and 80 years at the time of enrollment
7) Patients with sufficiently preserved systemic functions of the bone marrow, liver, kidneys, heart and lungs
8) ECOG performance status 0, 1 or 2
9) Preserved functions of major organs
10) No evidence of postoperative complications contraindicating the test treatment
11) Availability of informed consent from the patient for the study

0.4. Exclusion criteria

1) Patients with residual tumor of the surgical cut surface
2) Patients with active double cancer (synchronous double cancer or metachronous double cancer with a disease-free survival time of 5 years or less. Active double cancer excludes carcinoma *in situ* or lesions corresponding to cancer remaining in the mucosa for which local treatment can be viewed as curative.)
3) Patients with serious postoperative complications (postoperative infection, anastomotic failure, gastrointestinal bleeding, etc., which have not subsided by the time of registration)
4) Patients having any of the following complications

   Diabetes mellitus requiring insulin therapy or poorly controlled diabetes mellitus

   Poorly controlled hypertension

   History of myocardial infarction or unstable angina within the previous 6 months

   Liver cirrhosis

   Interstitial pneumonia, pulmonary fibrosis, severe pulmonary emphysema

5) Pregnant or lactating women
6) Patients having psychiatric disease or symptoms, whose participation in the clinical study is judged to be difficult
7) The investigator considers not suitable for the study

0.5. Study outline

Treatment

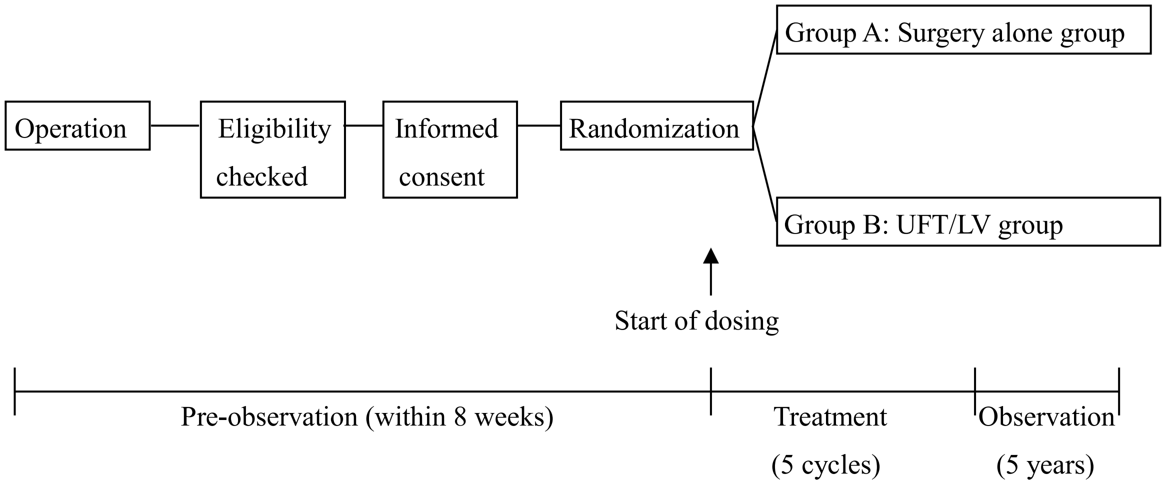

0.6. Planned number of subjects and study period

Estimated enrollment: 180 cases in total (90 cases/group)

Study period: January 1, 2004 to December 31, 2013 (end of registration: December 31, 2010)

Table of Contents

List of abbreviations

0. Summary i

1. Background 1

1.1 Target disease 1

1.2. Standard treatment for the target disease 2

1.3. Rationale for the target population selection 2

2. Objectives 3

2.1. Rationale for selection of the protocol treatment 3

2.2. Study significance 4

2.3. Benefits and risks (disadvantages) anticipated from the study 4

3. Test drug profile 5

4. Subjects 6

4.1. Inclusion criteria 6

4.2. Exclusion criteria 6

5. Method of consent acquisition 7

5.1. Consent for a comparative clinical study 7

5.2. Consent for measurement of the predictive factors of the responses to anticancer drugs 8

6. Study methods 8

6.1. Design 8

6.2. Outline 8

6.2.1. Surgery alone (Group A: Surgery alone group) 8

6.2.2. UFT/LV therapy (Group B: Adjuvant Treatment group) 9

6.3. Planned period of participation in the study 9

6.4. Test drug dosage & administration 9

6.5. Test drug formulation/composition, appearance, package, label and storage 10

6.6. Rules on concomitant therapy 10

6.7. Drug suspension and dose reduction 11

6.8. Information on drug intake advice 12

6.9. Methods of registration and allocation 12

７. Definition of study population and endpoints 14

7.1. Study population 14

7.2. Definition of Endpoints 14

8. Observation/laboratory test items and the follow-up period 15

9. Criteria for discontinuation of the protocol UFT/LV therapy 17

10. Dealing with adverse events 18

10.1. Dealing with the situation upon development of adverse events 18

10.2. Reporting of serious adverse events 18

11. Reporting any deviations from the protocol 19

12. Study completion, discontinuation and suspension 19

12.1. Completion 19

12.2. Discontinuation or suspension of the study 19

13. Study period 20

14. Data processing and statistical analysis 20

15. Target number of subjects and the rationale for setting it 20

16. Consideration of subjects' human rights, safety and disadvantages 20

16.1. Human rights 20

16.2. Safety/disadvantages 21

17. Expenses to be borne by patients 21

18. Health hazard compensation and insurance coverage 21

19. Compliance with GCP and Declaration of Helsinki 21

20. Storage of records 21

21. Publication of study results 22

22. Study organization 22

23. Research funds and conflicts of interests 22

24. Amendment of protocol, etc. 23

25. Reference information and papers 23

1. Background

1.1. Target disease

Since the report by Foster et al. [1], [illegible]sefulness of hepatectomy in the treatment of liver metastasis from colorectal cancer has been acknowledged widely, with reported resection and 5-year survival rates of 10 to 40% and 30 to 45%, respectively [2-8]. Along with the aggressive extension of surgical indications, long-term survival has been reported also among patients having unfavorable prognostic factors [9,10]. Meanwhile, the incidence of recurrence after hepatectomy is high, with the highest incidences of recurrence being reported in the residual liver (50%) and the lungs (20%). To date, however, sufficient evidence has not been collected to determine whether adjuvant chemotherapy should be administered or not for the prevention of recurrence and which route of administration would be suitable for adjuvant chemotherapy.

In Japan, prophylactic hepatic artery infusion therapy tends to be applied preferentially for the purpose of preventing recurrence in the residual liver, but no clear evidence is available on the effect of

such therapy in improving the prognosis of the patients [11]; furthermore, in view of the stress and the risk of complications arising from hepatic artery infusion therapy, we have to say that there is no clear evidence yet to support active promotion of such therapy. Recently, a two-arm comparative study comparing prophylactic hepatic artery infusion therapy and systemic chemotherapy was started and is now under way (Cooperative Project No.29: Japanese Foundation for Multidisciplinary Treatment of Cancer). However, registration of cases has not advanced smoothly, probably because of the high stress level in both groups despite the nature of the prophylactic chemotherapy.

According to a recent retrospective study [12], the recurrence-free survival rate after resection of liver metastasis from colorectal cancer was significantly higher in the prophylactic systemic chemotherapy group (mostly treated with an oral fluoropyrimidine preparation) than in the surgery alone group. In the analysis of the site of recurrence, the incidence of recurrence in the residual liver did not differ between the two groups, while that of recurrence in the lungs tended to be lower in the prophylactic systemic chemotherapy group. Overall, the data in the systemic chemotherapy group were even better than the data in the hepatic artery infusion group, suggesting that prevention of extrahepatic recurrence (recurrence in the lungs, etc.) may lead to a better outcome.

Because of the high frequency of recurrence in the residual liver after hepatectomy, close attention has been paid to drug infusion into the hepatic artery for the prevention of recurrence in the residual liver. However, in view of the relatively favorable outcome of proactive rehepatectomy in cases with recurrence in the residual liver and the effectiveness of hepatic artery infusion therapy in inoperable cases (treatment not aimed at prevention), we considered that it may now be necessary to review post-hepatectomy adjuvant chemotherapy from the viewpoint of preventing extrahepatic recurrence. To date, however, no such review has been carried out. Under these circumstances, we paid attention to UFT + oral leucovorin therapy, which was recently approved in Japan and has the potential of becoming a standard chemotherapy after resection of advanced colorectal cancer, and planned an investigator initiated clinical study aimed at examining whether or not this treatment could improve the prognosis of the patients after hepatectomy.

1.2. Standard treatment for the target disease

1) Hepatectomy

In patients treated by surgery alone, the reported 5-year survival rate is about 30 to 45%. Hepatectomy is indicated, regardless of the number and size of the metastatic foci, in cases satisfying all of the following criteria: (1) absence of metastasis in any organ other than the liver (e.g., lungs and lymph nodes); (2) the intrahepatic metastatic foci can be resected completely; (3) complete hepatectomy is possible, taking into consideration the reserves and status of the residual liver.

2) Rehepatectomy

Of all the patients undergoing complete resection of the intrahepatic metastatic foci during the first operation, 45 to 80% of patients develop recurrence (recurrence in the liver alone in about 30%). Surgical resection is the only means for potentially curative treatment of the recurrent tumor in the

residual liver. For this reason, surgical treatment is actively undertaken as rehepatectomy. If the tumor can be resected completely by rehepatectomy, prognosis similar to that after the first resection can be expected [13-16].

3) Hepatic artery infusion

Continuous hepatic artery infusion is applied to inoperable cases of liver metastasis. It is usually indicated in cases where the number of metastatic foci is ≥ 6-7 (as counted on CT images), or if the number of foci is smaller, but the metastatic focus is located close to the central Glisson's capsule or the root of the hepatic vein, with tumor invasion of vessels severe enough to make curative resection difficult even by lobectomy. 5-FU is continuously infused into the hepatic artery via a hepatic intra-arterial infusion pump or a disposable infuser system.

4) Systemic chemotherapy

Cases with metastatic foci outside the liver are considered as candidates for systemic chemotherapy. The standard regimen for systemic chemotherapy is combined 5-FU + LV therapy, which has been shown to be significantly superior in terms of the response rate to no treatment or 5-FU monotherapy. In Western countries, 5-FU + LV therapy combined with CPT-11 now represents standard therapy, but this regimen has not yet been established in Japan and is not considered as standard therapy.

1.3. Rationale for the target population selection

Recurrence occurs in 45 to 80% of all cases treated with complete resection of the metastatic foci during the first operation, with recurrence in the liver alone seen in about 30% of all cases. Although hepatic artery infusion therapy has been considered to be effective against recurrence in the residual liver, no conclusion has yet been reached as to whether or not suppression of recurrence in the residual liver might contribute significantly to prolongation of the survival time. Although second or third resection in cases of recurrence in the residual liver is sometimes technically difficult, the surgical stress associated with such resection is similar to that associated with the first resection.

To improve the outcome of treatment of colorectal cancer patients with liver metastasis, it is indispensable to improve the long-term survival rate. To this end, the effectiveness of systemic therapy for the control of extrahepatic lesions needs to be investigated.

2. Objectives

The study is aimed at evaluating the usefulness of postoperative adjuvant chemotherapy in colorectal cancer patients with liver metastasis undergoing macroscopically curative resection of the liver metastasis, through comparing the endpoints between patients undergoing hepatectomy alone ("the surgery alone group") and those treated by hepatectomy + UFT/LV therapy.

Primary endpoint: Recurrence-free survival

Secondary endpoint: Overall survival, safety

2.1. Rationale for selection of the protocol treatment

At present, the standard regimen for systemic chemotherapy for colorectal cancer is 5-FU + LV therapy. As stated above, a randomized comparative study showed non-inferiority of UFT/LV therapy to 5-FU + LV therapy, and UFT/LV therapy was approved in September 2003 in Japan. This therapy was adopted as the protocol treatment for this study.

UFT/LV Therapy

Dosage & Administration

Usually, adults are orally treated with folinate at a dose level 75 mg three times daily (at intervals of about 8 hours) together with the tegafur-uracil combination. The oral dose level of the tegafur-uracil combination is usually 300 to 600 mg (on a tegafur dose basis)/day (300 mg/m^2), administered in three divided doses (at internals of about 8 hours), avoiding administration within one hour before or after a meal.

These drugs are administered orally for 28 consecutive days, followed by a 7-day rest. The same sequence (one cycle) of treatment is then repeated every 5 weeks.

Efficacy data

In the bridging study conducted in Japan and the USA (involving 44 Japanese subjects), the response was CR in 2 cases and PR in 14 cases, corresponding to a response rate of 36.4% (16/44, 90% CI: 24.3-49.9).

Rationale for adopting the 'surgery-alone group' as the control group

In Western countries, 5-FU + leucovorin (5-FU + LV) therapy is acknowledged as the standard regimen for adjuvant chemotherapy after curative resection of primary colorectal cancer at Dukes B or C [17]. However, there is no report indicating the usefulness of 5-FU + LV after macroscopically radical resection of liver metastasis (Dukes D cases). Furthermore, serious adverse events to this therapy are seen at a rather high frequency of 15 to 20% [18], and this regimen has not been evaluated as prophylactic chemotherapy.

In 1999, leucovorin combined with fluorouracil was also approved in Japan for the treatment of colorectal cancer. However, the section "Important Precautions" in its package insert states: "In Japan, neither the efficacy nor the safety of this therapy as postoperative adjuvant chemotherapy has been established."

While the number of large-scale comparative studies involving a 'surgery-alone group' is small in Japan, a large-scale comparative study of the efficacy of UFT in Dukes B and C cases including a surgery-alone group is now under way (NSAS-CC), and the final results are awaited. With this background taken into account, the surgery-alone group was adopted as the control group for this

comparative study on adjuvant chemotherapy.

2.2. Study significance

This study is expected to: (1) yield clear evidence that adjuvant systemic chemotherapy after hepatectomy can improve the prognosis, and (2) allow adjuvant systemic chemotherapy to supplant hepatic artery infusion therapy, currently employed frequently in Japan for this indication, in order to reduce the stress and the risk of complications arising from prophylactic infusion into the hepatic artery. Furthermore, the study would enable evaluation of oral-dose anticancer drugs used frequently in Japan for the treatment of advanced/recurrent colorectal cancer. Thus, regardless of the results, positive or negative, this study is expected to be of high clinical significance.

2.3. Benefits and risks (disadvantages) anticipated from the study

Anticipated benefits

All the expenses for the drugs and care for the participating patients during the study will be borne by the national health insurance and the patients' co-payment. As a subsidy to cover the expenses for the visits to the hospital, the patients will receive 7000 Yen per visit (twice a month at the maximum) during the one-year period after discharge from the hospital. The subsidy shall also be paid for visits made to receive tests alone at the outpatient clinic.

Anticipated risks and disadvantages

In the Japan-USA bridging study that adopted a treatment schedule similar to that for the ULV treatment group in this study, diarrhea was the most frequent grade 3 or higher adverse event (9.1%) among the Japanese subjects in the UFT/LV group. To minimize the risks and disadvantages arising from adverse events, "4.1. Inclusion criteria, 6.6. Rules on concomitant therapy and 6.7. Drug suspension and dose reduction" have been carefully set for this study.

3. Test drug profile

See the package insert for UFT and UZEL Tablets/Leucovorin Tablets.

4. Subjects

Patients satisfying all of the inclusion criteria and not falling under any of the exclusion criteria listed below shall be eligible for the study.

4.1. Inclusion criteria

Patients satisfying all of the following requirements shall be considered as being eligible for the study.

1) Patients with histopathologically proven colorectal cancer with liver metastasis who have undergone surgical resection of the metastatic foci in the liver

2) Among the patients who have undergone macroscopically radical resection for foci of liver metastasis

[1] Cases confirmed by intraoperative ultrasonography to be free of unresected foci of liver

metastasis

[2] No evident cancer at the stump after hepatectomy (zero [0] mm is acceptable)

[3] Cases confirmed by palpation of hilar and paraaortic lymph nodes as having no metastasis in these nodes. Cases with evident metastasis in these lymph nodes will be excluded. (Sampled for histological examination as needed)

3) Patients undergoing treatment of liver metastasis for the first time or who have undergone hepatectomy once before. The liver metastasis may be synchronous or metachronous, but the period from resection of the primary tumor to diagnosis of the liver metastasis may not exceed 12 months. Patients undergoing rehepatectomy shall be eligible only if they were not registered for this study at the time of the first hepatectomy.

*There is no restriction depending on whether the first hepatectomy was carried out at own study institution or at another institution, the period from the first resection of liver metastasis to the rehepatectomy, or the presence/absence of adjuvant chemotherapy.

4) No extrahepatic lesions

[1] Confirmed by plain chest X-ray or CT to have no lung metastasis.

[2] In cases of metachronous liver metastasis, confirmed by barium study or colorectal endoscopy (or pelvic CT) to have no evidence of local recurrence.

[3] Confirmed by epigastric CT to have no enlarged lymph nodes in the hepatic hilus or the paraaortic region.

5) No previous history of treatment for hepatic metastasis (e.g., local/systemic chemotherapy or radiotherapy)

There is no restriction on chemotherapy received before detection of the first liver metastasis (e.g., adjuvant chemotherapy after surgery for colorectal cancer), but at least 3 months should have passed after the last drug dose of such chemotherapy.

6) Age between 20 and 80 years on the day of registration

7) Patients with sufficiently preserved systemic functions of the bone marrow, liver, kidneys, heart and lungs

8) Performance status 0, 1 or 2

9) Patients satisfying the following requirements at the start of treatment and thereby confirmed to have achieved sufficient recovery of the functions of organs after the surgery:

[1] WBC $\geq$ 4,000/mm^3 or $\leq$ 12,000/mm^3

[2] Platelet count $\geq$ 100,000/mm^3

[3] Hemoglobin $\geq$ 9.0 g/dl

[4] Serum total bilirubin $\leq$ 1.5 mg/dl

[5] AST $\leq$ 100 IU/l, ALT $\leq$ 100 IU/l

[6] Prothrombin activity $\geq$ 50%

[7] Serum creatinine $\leq$ 1.5 mg/dl

[8] BUN $\leq$ 25 mg/dl

[9] TP $\geq$ 5.9 g/dl

[10] Alb $\geq$ 3.0 g/dl

[11] CRP $\leq$ 2.1 ng/ml

10) No evidence of postoperative complications contraindicating the test treatment

11) Availability of informed consent from the patient for the study

4.2. Exclusion criteria

1) Patients with an evidently positive stump after hepatectomy
2) Patients with active double cancer (synchronous double cancer or metachronous double cancer with a disease-free survival time of 5 years or less. Active double cancer excludes carcinoma *in situ* or lesions corresponding to cancer remaining in the mucosa for which local treatment can be viewed as curative.)
3) Patients with serious postoperative complications (postoperative infection, anastomotic failure, gastrointestinal bleeding, etc., which have not subsided by the time of registration)
4) Patients having any of the following complications

   Diabetes mellitus requiring insulin therapy or poorly controlled diabetes mellitus

   Poorly controlled hypertension

   History of myocardial infarction or unstable angina within the previous 6 months

   Liver cirrhosis

   Interstitial pneumonia, pulmonary fibrosis, severe pulmonary emphysema
5) Pregnant or lactating women
6) Patients having psychiatric disease or symptoms, whose participation in the clinical study is judged to be difficult
7）Patients judged by the physician as being inappropriate for registration in the study

5. Method of Informed Consent

5.1. Consent for the comparative clinical study

The Investigator or the staff in charge of the study shall furnish each candidate patient with a leaflet carrying the necessary information to enable the patient's understanding and give a detailed explanation about the aspects of the study listed below, in writing as well as orally. As a rule, the explanation will be provided using the informed consent form (ICF) prepared by this study group (Attachment 1). If the ICF has been modified at any participating facility in accordance with the standards of the Institutional Review Board (IRB), the modified ICF needs to be sent to the Principal Investigator. The Principal Investigator shall check the modified ICF.

1. Introduction: Investigator initiated clinical study
2. Objectives of the study
3. Study methods

4. Planned period of your participation in the study
5. Planned number of participants
6. Effects anticipated and possible adverse events during this study
7. Other methods of treatment available
8. Upon outbreak of any health hazards
9. Participation in the study by the free will of individual patients
10. Supply of information about these drugs whenever available
11. Possibility of discontinuation of treatment with these drugs
12. Protection of the subjects' privacy at the time of publication of the study results
13. Instructions to follow after issuing consent for this study
14. Expenses to be borne by the subjects
15. Physician in charge
16. Consultation desk

Each candidate patient is requested to participate in the study; he/she shall be given the opportunity for making inquiries and a sufficient time to make the decision. When the patient gives consent for the study at his/her own discretion, the patient's name and the name of the explaining physician shall be entered together with the date of consent into the consent form prepared by the Secretariat or the approved consent form at the given facility.

The Investigator or the staff in charge shall hand a photocopy of the signed consent form to the patient. The original of the signed consent form will be stored at the participating facility.

If information on the efficacy, safety, etc., that can potentially affect the patient's consent for the study has been collected or if the protocol or the like is modified in a way that could affect the patient's consent, the relevant information shall be supplied immediately to the patient to confirm the patient's intention to participate in the study. Furthermore, the leaflet for consent acquisition, etc., will be modified in advance with the approval of the IRB and the consent of the patient will be obtained again.

5.2. Consent for measurement of the predictive factors of the responses to anticancer drugs

After sufficient understanding by the patient on providing samples for measurement of the predictive factors is obtained, explanation to gain the cooperation for the explorative study shall be given orally and in writing.

The explanation shall be provided in a simple manner so that patients can easily understand it, about the objectives and significance of the study, about the patient being at no therapeutic disadvantage in case he/she refuses to participate in the study, about the utmost care that will be taken to protect personal information about the patient, etc. After consent is obtained, the patient shall be asked to sign the consent form.

6. Study methods

6.1. Design

A randomized open-label clinical study involving parallel-group comparison with untreated controls and an explorative clinical study

6.2. Outline

After consent has been obtained from the patients eligible for the study, the patients shall be allocated at random to treatment Group A (surgery-alone group) or Group B (adjuvant therapy group), and the allocated treatment shall be started within 8 weeks after the surgery.

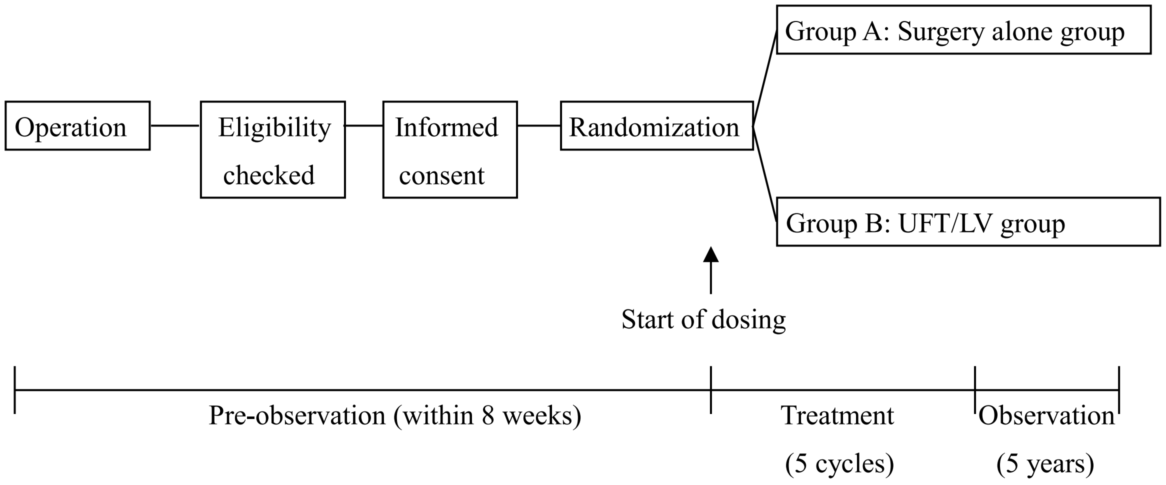

6.2.1. Surgery-alone (Group A: Surgery-alone group)

Patients allocated to the surgery-alone group shall be followed without treatment until detection of metastasis or recurrence. Follow-up examination at the outpatient clinic shall be carried out at least once in 4 months during the first year after the surgery and at least once in 6 months from the 3rd postoperative year onward.

6.2.2. UFT/LV therapy (Group B: Adjuvant Treatment group)

Treatment regimen

Chemotherapy shall be started within 8 weeks after the surgery, using the following regimen (5 weeks = 1 cycle) repeated for 5 cycles.

Drug	Dose	Route	Day of treatment
UFT	300 mg/m^2/day	p.o.	days 1-28
LV	75 mg/body/day	p.o.	days 1-28

Each treatment cycle consists of 28-day treatment with UFT + LV, followed by a 7-day rest.

Oral UFT dose level and dividing

Body surface area	Dose	Morning	Noon	Evening
1.17 ≤ BSA < 1.50	400	200	100	100
1.50 ≤ BSA < 1.84	500	200	200	100
1.84≤ BSA	600	200	200	200

1) The UFT dose level shall be determined as shown above on the basis of the body surface area.
2) No dose adjustments based on changes in the body weight shall be performed after the start of treatment.
3) UFT and LV shall be administered simultaneously three times daily.

6.3. Planned period of participation in the study

During the study, the medication shall be started within 8 weeks after the hepatectomy and continued for 25 weeks.

The registration period is 3 years. This period may be extended if the target number of subjects has not been registered in this period.

All the registered patients will be followed up until 5 years after registration of the last subject.

6.4. Test drug dosage & administration

Usually, adults are orally treated with folinate at a dose level 75 mg three times daily (at intervals of about 8 hours) together with the tegafur-uracil combination. The oral dose level of the tegafur-uracil combination is usually 300 to 600 mg (on a tegafur dose basis)/day (300 mg/m^2), administered in three divided doses (at internals of about 8 hours), avoiding administration within one hour before or after a meal.

These drugs are administered orally for 28 consecutive days, followed by a 7-day rest. The same sequence (one cycle) of treatment is then repeated every 5 weeks.

The total duration of the treatment shall be 25 weeks (5 cycles).

Reason for setting the UFT/LV therapy period at 6 months

In the Intergroup Protocol 0089 Study, the 5-FU + LV + LEV regimen (treatment for 6 months) was compared with 5-FU + LV (treatment for 6 months or 6 cycles of weekly treatment) and 5-FU + LEV (treatment for 12 months). There was no difference in terms of the recurrence-free survival time or overall survival time after 3.8 years of follow-up among the four regimens. From the viewpoint of toxicity, cost and convenience for the patient, the 6-month treatment with 5-FU + LV was judged to be superior to the 12-month treatment with 5-FU + LEV [18]. NCCTG and NCIC, on the other hand, reported a cooperative clinical study that was designed to evaluate the optimal period of postoperative adjuvant therapy for high-risk colorectal cancer patients (NCCTG 89-46-51) [19]. Comparison among

multiple postoperative chemotherapy regimens (5-FU + LEV and 5-FU + LV + LEV) administered for 6 or 12 months using a 2 x 2 factor design revealed that none of these regimens had any significant influence on the recurrence-free survival rate or the overall survival rate. However, the study revealed significant correlation between the treatment period and the regimen. Following this finding, each treatment group was separately analyzed, and the highest survival rate was found in the 6-month 5-FU + LV + LEV treatment group, although it was not significantly higher than the survival rate with any of the regimens for 12 months. These results suggest that 6-month 5-FU + LV treatment is comparable to or superior to 12-month treatment with 5-FU + LEV or 5-FU + LV in terms of the efficacy. This regimen is thus considered as the standard regimen at present.

Regarding UFT/LV therapy, on the other hand, the Intergroup CA-011 Study (primary endpoint: overall survival) [20] and the Intergroup CA-012 Study (primary endpoint: time to progression) [21] demonstrated non-inferiority of UFT/LV to 5-FU + LV therapy. Thus, although indirectly, it was indicated that it would be rational to set the same treatment period for this therapy as that for 5-FU + LV therapy.

6.5. Test drug formulation/composition, appearance, package, label and storage

See "**3. Test drug profile**" for the profile of each drug involved in folinate/tegafur-uracil therapy.

6.6. Rules on concomitant therapy

Prohibited concomitant drugs

Tegafur-gimeracil-oteracil potassium combination (TS-1) may not be used concomitantly because of the risk of appearance of serious adverse events (myelosuppression, etc.) when used together with test drugs.

Concomitant therapy/supportive therapy not acceptable with UFT/LV therapy

Anticancer drugs other than UFT, BRM (Picibanil, Lentinan, Krestin), radiotherapy

Concomitant drugs requiring exercise of caution

Drug interactions (care is needed in concomitant use)

1) Phenytoin

Signs of phenytoin intoxication (dyslalia, ataxia, disturbance in consciousness, etc.) may appear. The blood level of phenytoin may rise although the mechanism and risk factors are unknown.

2) Warfarin potassium

The activity of warfarin potassium may be reinforced although the mechanism is unknown. Therefore, attention must be paid to changes in the blood coagulability.

6.7. Drug suspension and dose reduction

Rules on modification of UFT/LV therapy

1) Requirements for patients receiving the treatment

[1] Tests conducted on the planned day of start of each treatment course or on the day of visit during the course of treatment should satisfy all of the following criteria:

WBC ≥ 4,000/mm^3, platelet count ≥ 100,000/mm^3

Serum total bilirubin ≤ 1.5 mg/dl, AST (GOT) < 100 IU/l, ALT (GPT) < 100 IU/l

[2] All non-hematological toxicities other than constipation, AST (GOT), ALT (GPT), total bilirubin and alopecia should be grade 1 or lower.

2) Rules for postponement/suspension

i) Treatment shall be administered to cases satisfying all of the above-mentioned requirements at the start of a given course of treatment. If any of the requirements is not satisfied, the treatment shall be postponed until the criteria are satisfied. Treatment shall not be skipped.

ii) If any of the above-mentioned requirements is not satisfied during the course of treatment, treatment will be suspended. If the check carried out a week later reveals complete recovery and satisfaction of the criteria, treatment shall be resumed. Upon resumption, treatment shall be continued from where it was left off at the time-point of suspension, without skipping any of the planned days of treatment.

iii) If treatment cannot be resumed within 15 days after postponement or suspension, the protocol treatment will be discontinued.

3) Rules on dose reduction

	Standard		-1 level	
Body surface area	Dose	Morning-Noon-Evening	Dose	Morning-Noon-Evening
1.17 ≤ BSA < 1.50	400 mg	200-100-100	300 mg	100-100-100
1.50 ≤ BSA < 1.84	500 mg	200-200-100	400 mg	200-100-100
1.84 ≤ BSA	600 mg	200-200-200	500 mg	200-200-100

When any of the conditions listed below in i) through iii) occurs during the course of treatment, dose reduction shall be carried out. Once the -1 level has been adopted, the dose level shall not be increased again even when the signs of toxicity have disappeared. If the same signs of toxicity are seen also at the -1 dose level, the protocol treatment shall be discontinued.

i) In case of WBC < 1,000/μl or PLT< 25,000/μl, the -1 dose level shall be used after the requirements for patients receiving the treatment are met again.

ii) If Grade 3 non-hematological toxicity is seen, treatment at the -1 dose level shall be resumed in the next course of treatment after alleviation of each sign of toxicity to Grade 1 or lower.

iii) If the requirements for patients receiving the treatment are satisfied again between 9 and 15 days after postponement or suspension, treatment shall be resumed at the -1 dose level.

6.8. Information on drug intake advice

The drugs should be taken in accordance with the dosage & administration for “folinate/tegafur-uracil therapy” described in “**3. Test drug profile**.”

6.9. Methods of registration and allocation

To avoid biases in the important prognostic factors among the treatment groups, the patients shall be allocated at random by the minimization algorithm involving the following factors.

Factor	Level
Institution	University of Tokyo, Cancer Institute Hospital of JFCR, NTT Medical Center Tokyo, Toranomon Hospital, Sempos Tokyo Takanawa Hospital, Showa General Hospital, Yokohama Seamen's Insurance Hospital, Social Insurance Chuo General Hospital, Tokyo Metropolitan Hiroo Hospital, Nihon University, Juntendo University, Shinshu University, JR Tokyo General Hospital
Primary site	colon/rectum
Timing	synchronous (DFI<1yr) /metachronous
No. of Tumor	single / multiple
Hepatectomy	first / second

1) The physician in charge, after confirming acquisition of informed consent, shall access the Homepage prepared by UMIN, enter the necessary information and perform allocation, and thus register the patients into the groups presented.
2) The UMIN Homepage for allocation can be accessed only by a pre-registered user (investigator).
3) The physician in charge shall enter the registration number into the registration confirmation form and begin the treatment.

Contact for case registration:

Secretariat (Physician's Office, Hepato-Biliary-Pancreatic Surgery Division, University of Tokyo)
Telephone: +81-3-5800-8654　FAX: +81-3-5800-8844
UMIN Homepage for allocation: https: //center.umin.ac.jp/islet/lvuft/

Inquiry about inclusion criteria:

Norihiro Kokudo, Kiyoshi Hasegawa
Hepato-Biliary-Pancreatic Surgery Division, University of Tokyo
Telephone: +81-3-5800-8654
e-mail: KOKUDO-2SU@h.u-tokyo.ac.jp

7. Definition of the study population and endpoints

Primary endpoint: Recurrence-free survival

Secondary endpoint: Overall survival, safety

7.1. Study population

Total registered patients

"Total registered patients" is defined as all patients registered in accordance with the registration procedure.

Total eligible patients

"Total eligible patients" is defined as all registered patients excluding patients who are judged as "ineligible" by review within the group.

Total treated patients

"Total treated patients" is defined as the total number of registered patients who have received a part or the whole of the protocol treatment.

7.2. Definition of Endpoints

1) Recurrence-free survival: Period from the date of randomization to detection of recurrence. For patients who have undergone rehepatectomy, it would be the period from the date of randomization followed by rehepatectomy to detection of the second recurrence. Recurrence or death from underlying disease, whichever occurs the earliest, shall be counted as the event, whereas death from other diseases without recurrence shall be as the censor. In the other patients, the day of final confirmation of recurrence shall signal the end of this period. The cumulative recurrence-free survival rate will be calculated by Kaplan-Meier method. Comparison of the recurrence-free survival shall be conducted using the log-rank test. Analysis using the Cox regression model shall be performed in addition.
2) Overall survival: Period from the date of randomization to death. For patients who have undergone rehepatectomy, this will be the period from the date of randomization followed by rehepatectomy to death. Death from underlying disease or death from other disease shall be counted as the event. For survivors and patients for whom the survival/death status is unknown, the last point of time at which survival is confirmed shall signal the end of this period. The cumulative survival rate will be calculated by Kaplan-Meier method. Comparison of the overall survival shall be carried out using the log-rank test. Analysis using the Cox regression model shall be performed in addition.

8. Observation/laboratory test items and the follow-up period

1) Background variables Birth date, date of operation, height, body weight
2) Surgical/histological findings
3) mRNA expression levels of TS, DPD, TP, FPGS, GGH, DHFR, ERCC1, Topo1, EGFR, VEGF, etc., in the surgical specimens
4) Status of medication (drug suspension, dose reduction, etc.)
5) Compliance with dosing instructions: Checked by interview during each outpatient clinic visit

6) Laboratory tests and subjective/objective findings

In accordance with the evaluation schedule given in the table below, hematology/biochemistry/urinalysis and tumor marker examinations shall be performed and the clinical findings checked at 3, 6, 9 and 12 months, at 1 year 6 months, and at 2, 3, 4 and 5 years after surgery. Diagnostic imaging shall be performed at 6 months, 1 year (± 1 month), 1 year 6 months, 2, 3, 4 and 5 years (± 3 months) after surgery.

[1] Laboratory tests:

[Tests]

Hematology: WBC, Plt, Hb

Biochemistry: AST, ALT, total bilirubin, ALP, BUN, Creatinine

Urinalysis: protein, glucose, occult blood

Tumor markers: CEA, CA19-9

[2] Subjective/objective findings: Checked by interview during the outpatient clinic visit.

Anorexia, nausea/vomiting, diarrhea, stomatitis, eruption, vertigo/dizziness, dysgeusia, peripheral neuropathy, alopecia, etc.

(Notes)

・In the UFT/LV group, laboratory testing shall be conducted periodically at least once during one cycle (5 weeks) of treatment (particularly in the first two cycles of treatment, the tests shall be conducted at the start of each cycle and then at least once after the start of each cycle) to ensure the safety of the patients.

・In view of possible appearance of serious liver disorder (e.g., fulminant hepatitis), adequate care shall be paid to any malaise accompanied by anorexia, etc., which may be viewed as precursory subjective symptoms of liver disorder.

・In view of possible appearance of serious enteritis or signs of dehydration, close attention shall be paid to intense abdominal pain, diarrhea, etc.

Evaluation schedule

	Hematology, Biochemistry, Urinalysis, Clinical findings	Diagnostic imaging		Tumor markers CEA, CA19-9
		Abdominal US	Abdominal and thoracic CT	
Registration	○			
(After surgery) 1 month				○
2 months				○
3 months	○	○		○
4 months				○
5 months				○
6 months	○	○	○	○

7 months				○
8 months				○
9 months	○	○		○
10 months				○
11 months				○
1 year	○	○	○	○
1 year 2 months				○
1 year 4 months				○
1 year 6 months	○	○	○	○
1 year 8 months				○
1 year 10 months				○
2 years	○	○	○	○
2 years 2 months				○
2 years 4 months				○
2 years 6 months				○
2 years 8 months				○
2 years 10 months				○
3 years	○	○	○	○
3 years 6 months				○
4 years	○	○	○	○
4 years 6 months				○
5 years	○	○	○	○

7) Adverse events: Any accompanying symptoms and changes in laboratory parameters whose causal relationship to UFT/LV therapy cannot be ruled out are counted as adverse events. The nature of the adverse events, its severity, date of appearance, measures taken to control the reaction (symptomatic treatment, drug suspension, discontinuation, etc.), the clinical course, the outcome, causal relationship to the test treatment (1. Related, 2. Possibly related 3. Not related), etc., shall be entered in the survey form.

8) Definition of recurrence

[1] Recurrence shall be identified by diagnostic imaging (CT, US, bone scintigraphy, PET).

[2] Recurrence may also be diagnosed if the abdominal/thoracic CT, abdominal ultrasonography and bone scintigraphy (PET) are negative, but tumor marker levels show increase to levels exceeding 10 times the upper limit of the normal range prior to hepatectomy.

9) Checking for presence/absence and timing of recurrence

Presence/absence of recurrence is checked by measurement of the serum tumor marker levels (CEA, CA19-9; every other month), abdominal ultrasonography and plain chest X-ray (every 3rd month), abdominal and thoracic CT (every 6th month), barium study or colorectal endoscopy (or pelvic CT) (every 12th month), etc. Bone scintigraphy (PET) and brain CT may be skipped if there is no sign of metastasis. In cases with suspected recurrence, precise testing will be conducted, as needed, to check the site of

recurrence, the status of recurrence, etc. In cases where recurrence has been confirmed by diagnostic imaging, histological examination, etc., the day of such confirmation shall be counted as the day of recurrence.

10) Checking for survival/death and timing

Checking for survival/death shall be carried out every year until the end of the follow-up period. In the cases of death, distinction shall be made among death from cancer, death from other cancer, death from other disease.

9. Criteria for discontinuation of the protocol UFT/LV therapy

Definition of protocol treatment completion

Cases in which 5 courses of treatment have been completed

Criteria for discontinuation of the protocol treatment

1) If recurrence has been detected

Post-study treatment for the surgery alone group and the UFT/LV therapy group

In cases where recurrence has been confirmed, treatment shall be switched to the method judged as the best treatment method, taking into account the status of the metastatic foci, after giving sufficient information to the patient and confirming his/her understanding of the information. The details of the treatment provided needs to be entered in the case report form.

In cases of recurrence in the residual liver alone, rehepatectomy shall be performed as far as possible, and for inoperable cases, hepatic artery infusion therapy will be considered.

2) If the protocol treatment cannot be continued owing to the appearance of adverse events, as follows:

[1] Grade 4 non-hematological toxicity (non-hematological toxicity: adverse events other than NCI-CTC “blood/bone marrow”)

[2] Inability to resume treatment within 15 days after postponement or suspension of treatment because of toxicity

[3] Necessity for further dose reduction (beyond -1 level) due to toxicity

[4] Treatment discontinuation proposed by the physician in charge due to the occurrence of adverse events

3) If the patient requests discontinuation of the protocol treatment for reasons related to adverse events
4) If the patient requests discontinuation of the protocol treatment for reasons not related to adverse events
5) Death during protocol treatment
6) Cases found to have violated the protocol or to be ineligible to the study
7) Cases found to be pregnant
8) Other cases judged by the physician to require discontinuation of the study for any reason

Note 1: If the study is discontinued for the reasons specified in 2), the patient needs to be followed until restoration of the original condition as far as possible.

Note 2: The reason for the study discontinuation needs to be entered in the medical records and in the case report form.

Note 3: If consent for the study is withdrawn after the start of the test drug treatment, it needs to be clarified whether or not the consent withdrawal is due to lack of response to the test drugs, development of adverse events, or other accidental reasons (change of residence, etc.), so as to obtain information useful for judging the acceptability of inclusion of the patient in the analysis of the efficacy and safety.

10. Dealing with adverse events

10.1. Dealing with the situation upon development of adverse events

Upon development of an adverse event, the Investigator or Sub-investigator is required to take appropriate actions immediately, accompanied by entry of information about the event into medical records and the case report form in a consistent manner. If the test drug treatment has been discontinued or if treatment of adverse events has become necessary, the concerned subjects also need to be informed.

10.2. Reporting of serious adverse events

1) Definition of serious adverse events

- ・Death or events potentially leading to death
- ・Hospitalization or prolongation of hospital stay
- ・Disabilities or events potentially leading to disabilities
- ・Illness or anomaly in later generations or congenital illness or anomaly

2) Reportable adverse events

・All serious adverse events encountered during the study and any serious adverse events with possible causal relationship to the test drugs developing after completion (discontinuation) of the study need to be reported.

・Upon development of a serious adverse event, the Investigator is required to report it immediately to the Hospital Director and to the Clinical Study Department of the hospital. If the study involves multiple facilities, such information also needs to be provided to the principal investigator of this study. The reports needed are the first report (expedited report) and the second report (detailed report).

・The reports need to be submitted within 7 or 15 days after the event depending on the seriousness and the category (known/unknown) of the event in accordance with Article 66-7 of the Ordinance for Enforcement of the Pharmaceutical Affairs Law. The facilities participating in this study are required to submit the reports in accordance with the set rules and format at individual facilities.

3) Reporting of other adverse events

・Other adverse events will be entered into the case report form in accordance with the procedure set forth in " 8. Observation/laboratory test items."

11. Reporting of any deviations from the protocol

1) The Investigator or Sub-investigator may not enforce any deviation from or amendment of the protocol without prior agreement of the Principal Investigator and prior authorization by the Hospital Director based on a review by the IRB.
2) If it is inevitable for any reason (e.g., emergency avoidance, etc.), the Investigator or Sub-investigator may enforce deviation from or amendment of the protocol without prior agreement of the Principal Investigator or prior authorization of the IRB. In such cases, the Investigator or Sub-investigator is required to submit a report on the deviation or amendment together with the reason and a draft amendment of the protocol (if amendment of the protocol, etc., is needed) to the Principal Investigator and the IRB and to get approval from the Principal Investigator, IRB and the Hospital Director.
3) If there is deviation from the protocol, the Investigator or Sub-investigator is required to take records of the deviation together with the reasons and to submit a report on the deviation in the form specified at the facility to the Hospital Director and the Principal Investigator. The photocopy of each of these documents needs to be retained by the Investigator.

12. Study completion, discontinuation and suspension

12.1. Completion

The study at a given participating facility shall be deemed to be completed at the end of the 5-year follow-up period of all the registered patients. The overall study shall be deemed to be completed when the study has been completed at all the participating facilities. Upon completion of the study at individual facilities, the Investigator is required to immediately submit the study completion report to the Hospital Director and the Principal Investigator.

12.2. Discontinuation or suspension of the study

In the following cases, the Investigator is required to judge whether or not the study should be continued. If discontinuation or suspension of the study is decided, the decision and the reason need to be immediately reported in writing to the Hospital Director.

1) If important information related to the quality, safety or efficacy of the test drugs has been obtained
2) If achieving registration of the target number of subjects is judged to be difficult due to difficulties in subject recruitment
3) If the objectives of the study are achieved before completion of registration of the planned number of subjects or elapse of the planned period
4) If amendment of the protocol, etc., is recommended by the IRB and it is considered difficult to accept the recommendation
5) If the IRB has ordered/recommended discontinuation of the study

13. Study period

January 1, 2004 to December 31, 2013

(End of registration: December 31, 2010)

Note 1: Period from consent acquisition from the first subject to the day of last observation of the last subject.

14. Data processing and statistical analysis

During the data processing, the data on the items set forth in the protocol are entered into the Case Report Form (CRF) for this study. The CRFs of individual subjects shall be collected at 12, 24, 36, 48 and 60 months after the surgery. The methods of analysis of the individual items are specified in "**7.2. Definition of endpoints**."

15. Target number of subjects and the rationale for setting it

Target number of subjects: 180 cases in total (90 cases/group)

When the expected number of patients eligible for this study at each participating facility was investigated in advance, it was estimated that 180 cases could be registered during the 3-year registration period. In view of this estimation, the number of subjects needed for this study was set at 90 cases/group and 180 cases in total, if the registration period were to be 3 years and the follow-up period were to be 3 years. In this case, the statistical power at a significance level of 5% (two-tailed) was determined to be about 75%, assuming a 3-year recurrence-free survival rate in the surgery alone group of 20% and an anticipated recurrence-free survival rate in the ULV/LV group of 35% (relative decrease = 0.35).

16. Consideration of subjects' human rights, safety and disadvantages

16.1. Human rights

When raw data, consent form, etc., related to the study are handled, adequate care will be taken to protect the subjects' privacy. The case reports, etc., submitted outside the hospital will carry only the subject ID code, etc. When the results of the study are to be published, any information allowing identification of the subjects will be erased. The data collected from the subjects during the study will not be used for any purpose other than this study.

Anonymization of personal information during exploration of the response-predictive factors will be ensured at each participating facility, and the identifiable information of individuals will be managed at each facility. At the collaborative research institutions outside the participating medical facilities, identification of the healthcare information will rely only on the ID code.

16.2. Safety/disadvantages

To minimize the risks of adverse events and disadvantages, "**4. Subjects**," "**4.1. Inclusion criteria**," "**4.2.Exclusion criteria**" and "**6.7. Drug suspension and dose reduction**" were set carefully.

Upon development of adverse events, appropriate checks and actions will be executed immediately. Further developments of the event will be prevented in accordance with "**10. Dealing with adverse events**."

17. Expenses to be borne by patients

All the drugs used in this study are covered by the national health insurance in Japan, and the same methods of treatment as those employed during routine insured healthcare services will be employed in this study. All of the expenses for the drugs and care of the participating patients during the study will be borne by the national health insurance and the patients' co-payment. As a subsidy to cover the expenses incurred by the hospital visits, the patients will receive 7000 Yen per visit (twice a month at the maximum) for a one-year period after discharge from the hospital. The subsidy will also be paid for visits made to receive tests alone at the outpatient clinic. No expense for any specific test is needed for the participants of this study.

18. Health hazard compensation and insurance coverage

Because commercially available drugs will be used in this study in accordance with their approved clinical dosage and administration route, the expenses for the treatment of any health hazards related to this study will also be borne by the national health insurance and the patients' co-payment, similar to the expenses for routine clinical care. The expenses for the treatment of adverse events arising in the registered patients will not be covered by public subsidies. The Investigator and Sub-investigator shall be covered by liability insurance in preparation for payment of damages.

19. Compliance with GCP and Declaration of Helsinki

This study shall be carried out in accordance with GCP and in compliance with the Declaration of Helsinki.

20. Storage of records

The Investigator is required to store the essential documents for implementation of this study, etc., listed below, and to dispose of them 5 years after publication of the study results.

・Photocopy of application documents
・Photocopy of applications/reports
・Consent forms
・Notifications from the Hospital Director
・List of subject ID codes
・Photocopy of case report forms, etc.

21. Publication of the study results

At the end of the follow-up period, the data from the final follow-up examination shall be fixed and analyzed as to all the endpoints. The results of the final analysis shall be summarized at the Hepato-Biliary-Pancreatic Surgery Division as the "final analysis results," and presented at the report meeting of all investigators engaged in this study and then published at professional society meetings and in professional journals.

22. Study organization

Organization for the study

Hepato-Biliary-Pancreatic Surgery Division, University of Tokyo

Colon and Rectal Surgery Division, University of Tokyo

Other participating facilities (as of May 2009) (in random order):

Akio Saiura Department of Gastroenterological Surgery, Cancer Institute Hospital of Japanese Foundation for Cancer Research, Ariake Hospital

Nobuyuki Mizunuma Department of Gastroenterology internal medicine, Cancer Institute Hospital of Japanese Foundation for Cancer Research, Ariake Hospital

Hiroto Koyama	Department of Surgery, Sempos Tokyo Takanawa Hospital
Masanori Teruya	Department of Surgery, Showa General Hospital
Yasutsugu Bandai	Department of Surgery, Social Insurance Chuo General Hospital
Shinichi Miyakawa	First Department of Surgery, Shinshu University
Seiji Kawasaki	Department of Hepato-Biliary-Pancreatic Surgery, Juntendo University
Tadatoshi Takayama	Department of Digestive Surgery, Nihon University
Masatoshi Makuuchi	Department of Surgery, Japanese Red Cross Medical Center
Fuyo Yoshimi	Department of Surgery, Ibaraki Prefectural Central Hospital and Cancer Center
Junji Yamamoto	Department of Surgery, National Defense Medical College

Consultant

Kiyohiko Hatake	Cancer Institute Hospital of Japanese Foundation for Cancer Research, Ariake Hospital
Yutaka Matsuyama	Department of Biostatistics, University of Tokyo

23. Research funds and conflicts of interest

Planning, implementation or reporting of this study shall involve no “possible conflict of interest” that could affect the results of this study or the interpretation of its results. Implementation of the study shall not injure the rights or benefits of any subject.

24. Amendment of protocol, etc.

Amendment of the protocol and changes/revision of the leaflet for consent acquisition require prior approval of the IRB or the ethics committee of each participating medical facility. If the IRB or the ethics committee demands amendment of the protocol for approval, the Investigator could modify the protocol at the medical facility concerned under agreement with the Principal Investigator.

25. Reference information and papers

1. Foster and Berman. 1977 Book, "Solid liver tumors"

2. Hughes et al. 1986 Surgery 100: 178-184

3. Nordlinger et al. 1987 Ann Surg 205: 256-263

4. Scheele et al. 1990 Br J Surg 77: 1241-1246

5. Doci et al. 1991 Br J Surg 78: 797-801

6. Sugihara et al. 1993 Br J Surg 80: 1032-1035

7. Fong et al. 1999 Ann Surg 230: 309-318

8. Kokudo et al. 2001 Am J Surg 181: 153-159

9. Minagawa et al. 2000 Ann Surg 231: 487-499

10. Weber et al. 2000 Ann Surg Oncol 7: 643-650

11. Takeo Mori et al. 2001 Jpn J Gastroenterol Surg 24: 313-319

12. Kokudo et al. 1998 Ann Surg Oncol

13.The American Journal of Surgery181(2001)153-159 etc etc

14.ANNALS OF SURGERY Vol.225.No.1,51-62

15.THE SURGICAL ONCOLOGY CLINICS OF NORTH AMERICA Vol.5.N.2.APRIL1996

16.]Jpn J Cancer Chemother 29(6): 848-855,June,200

17. Lancet 345: 939,1995

18. O'Connell et al.1997 J.Clin Oncol 15 : 246-250

19. O'Connell et al.2001 Proc Am Soc Clin Oncol 20 : 132a

20. Douilard J-Y et al.2002, J Clin Oncol 20 : 3605-16

21. Carmichael J et al. 2002 J Clin Oncol

22. Leichman, C.G. et al.,J.Clin.Oncol.,15: 3223-3229,1997

23. Lenz.H.J.et al., Clin.Cancer Res.,4: 1243-1250,1998

24. Cascinu.S.et al.,Clin. Cancer Res.,5: 1996-1999,1999

25. Bathe, O.F. et al., Cancer J Sci.Am.,5 : 34-40,1999

26. Paradiso,A. et al.,Br.J.Cancer 82 : 560-567,2000

27. Metzger,R. et al., Clin. Cancer Res., 4 : 2371-2376,1998

28. Saito,S. et al., Cancer, 88 : 42-49,2000

29. Etienne,M.C. et al.,J. Clin. Oncol.,13 : 1663-1670-1995

30. Salonga,D. et al.,Clin.Cancer Res.,6 : 1322-1327,2000

31. Odin, E. et al., Clin Cancer Res. 9: 6012-6019, 2003

32. Satta, T. et al., Jpn J Cancer Res. 82: 593-598, 1991

33. Shirota, Y. et al., J Clin Oncol; 19: 4298–4304, 2001

34. Paradiso, A.V. et al., Proc Am Soc Clin Oncol: abstr 599, 2001

35. Neal, D.E. et al., Lancet 1: 366-368, 1985

36. Cooper, B.C. et al., Clin Cancer Res. 8: 3193-3197, 2002

37. Zebrowski, B.K,, Ann Surg Oncol. 6: 373-378, 1999

38. Tamsma, J.T. et al., Ann Oncol. 12: 1353-1357, 2001

39. Hyodo, I. et al., Eur J Cancer. 34: 2041-2045, 1998

40. Takiuchi, H. et al., Oncol Rep. 7: 841-846, 2000
