# Supplementary material for: Adjuvant Oral Uracil-Tegafur with Leucovorin for Colorectal Cancer Liver Metastases: A Randomized Controlled Trial
Source: PLoS One. 2016 Sep 2;11(9):e0162400. doi: 10.1371/journal.pone.0162400 (PMC5010179; doi:10.1371/journal.pone.0162400)
Supplement: S3 File — (PDF) [file pone.0162400.s003.pdf]

List of Modifications of the Protocol for "Study of the Efficacy of Post-Hepatectomy Adjuvant Chemotherapy in Colorectal Cancer Patients with Liver Metastasis" (8th version)

No.	Page	Part	Before modification	After modification	Date of modification
1	p.i<br>p.5	0.3. Inclusion criteria<br>4.1. Inclusion criteria	3) Patient undergoing treatment of liver metastasis for the first time. The liver metastasis may be synchronous or metachronous, but the period from resection of the primary tumor to diagnosis of the liver metastasis may not exceed 12 months.<br><br>6) Age between 20 and 75 years old on the day of registration	3) Patients undergoing treatment for liver metastasis for the first time or who have undergone hepatectomy once before. The liver metastasis may be synchronous or metachronous, but the period from resection of the primary tumor to diagnosis of the liver metastasis may not exceed 12 months. Patients undergoing hepatectomy for the second time shall be eligible only if they were not registered for this study at the time of the first hepatectomy.<br>*There is no restriction depending on whether the first hepatectomy was carried out at own study center or at another facility, the period from the first resection of liver metastasis to the second hepatectomy, or the presence/absence of adjuvant chemotherapy. However, Criterion 5) of Section 0.3. needs to be satisfied.<br><br>6) Age between 20 and 80 years old on the day of registration	April 14, 2005<br>(3th version)
2	p.12	6.9. Methods of registration and allocation	Factor / Level<br>Institution: University of Tokyo, Cancer Institute Hospital of JFCR, NTT Medical Center Tokyo, Toranomon Hospital, Sempos Tokyo Takanawa Hospital, Showa General Hospital, Yokohama Seamen's Insurance Hospital, Social Insurance Chuo General Hospital, Tokyo Metropolitan Hiroo Hospital<br>Primary site: colon/rectum<br>Timing: synchronous (DFI<1yr)/metachronous<br>No. of Tumor: single/multiple	Factor / Level<br>Institution: University of Tokyo, Cancer Institute Hospital of JFCR, NTT Medical Center Tokyo, Toranomon Hospital, Sempos Tokyo Takanawa Hospital, Showa General Hospital, Yokohama Seamen's Insurance Hospital, Social Insurance Chuo General Hospital, Tokyo Metropolitan Hiroo Hospital, Nihon University, Juntendo University, Shinshu University, JR Tokyo General Hospital<br>Primary site: colon/rectum<br>Timing: synchronous (DFI<1yr)/metachronous<br>No. of Tumor: single/multiple<br>Hepatectomy: first/second	
3	p.14	7.2. Definition of endpoints	1) Recurrence-free survival: Period from surgery to detection of recurrence. Recurrence in the liver, death from the underlying disease or death from other diseases, whichever occurs the earliest, shall be counted as the event. In the other patients, the day of final confirmation of recurrence shall signal the end of this period. The cumulative recurrence-free survival rate will be calculated by Kaplan-Meier method. Comparison of the recurrence-free survival shall be conducted using the log-rank test. Analysis using the Cox regression model shall be performed in addition.<br>2) Overall survival: Period from surgery to death. Death from underlying disease or death from other disease shall be counted as the event. For survivors and patients for whom the survival/death status is unknown, the last point of time at which survival is confirmed shall signal the end of this period. The cumulative survival rate will be calculated by Kaplan-Meier method. Comparison of the overall survival shall be carried out using the log-rank test. Analysis using the Cox regression model shall be performed in addition.	1) Recurrence-free survival: Period from surgery to detection of recurrence. For patients who have undergone rehepatectomy, it would be the period from rehepatectomy to detection of recurrence. Recurrence in the liver, death from the underlying disease or death from other diseases, whichever occurs the earliest, shall be counted as the event. In the other patients, the day of final confirmation of recurrence shall signal the end of this period. The cumulative recurrence-free survival rate will be calculated by Kaplan-Meier method. Comparison of the recurrence-free survival shall be conducted using the log-rank test. Analysis using the Cox regression model shall be performed in addition.<br>2) Overall survival: Period from surgery to death. For patients who have undergone rehepatectomy, this will be the period from rehepatectomy to death. Death from underlying disease or death from other disease shall be counted as the event. For survivors and patients for whom the survival/death status is unknown, the last point of time at which survival is confirmed shall signal the end of this period. The cumulative survival rate will be calculated by Kaplan-Meier method. Comparison of the overall survival shall be carried out using the log-rank test. Analysis using the Cox regression model shall be performed in addition.	
4	p.iii<br>p.21	0.6. Planned number of subjects and study period<br>14. Study period	January 1, 2004 to December 31, 2009<br>(End of registration: December 31, 2006)	January 1, 2004 to December 31, 2011<br>(End of registration: December 31, 2008)	April 25, 2006<br>(4th version)
5	p.iii<br>p.21	0.6. Planned number of subjects and study period<br>14. Study period	January 1, 2004 to December 31, 2011<br>(End of registration: December 31, 2008)	January 1, 2004 to December 31, 2012<br>(End of registration: December 31, 2009)	April 3, 2008<br>(5th version)
6	p.iii<br>p.21	0.6. Planned number of subjects and study period<br>14. Study period	January 1, 2004 to December 31, 2012<br>(End of registration: December 31, 2009)	January 1, 2004 to December 31, 2013<br>(End of registration: December 31, 2010)	May 21 2009<br>(6th version)
